# Novel Histone Deacetylase (HDAC) Inhibitor Induces Apoptosis and Suppresses Invasion via E-Cadherin Upregulation in Pancreatic Ductal Adenocarcinoma (PDAC)

**DOI:** 10.3390/ph17060752

**Published:** 2024-06-07

**Authors:** Katja Schiedlauske, Alina Deipenbrock, Marc Pflieger, Alexandra Hamacher, Jan Hänsel, Matthias U. Kassack, Thomas Kurz, Nicole E. Teusch

**Affiliations:** 1 Institute of Pharmaceutical Biology and Biotechnology, Heinrich-Heine-University Düsseldorf, Universitätsstr. 1, 40225 Düsseldorf, Germany; 2 Institute of Pharmaceutical and Medicinal Chemistry, Heinrich-Heine-University Düsseldorf, Universitätsstr. 1, 40225 Düsseldorf, Germany

**Keywords:** pancreatic cancer, PDAC, histone deacetylase inhibitor, HDAC 2, HDAC 6, E-cadherin, apoptosis, invasion, EMT

## Abstract

Pancreatic ductal adenocarcinoma (PDAC) is the most lethal form of pancreatic cancer characterized by therapy resistance and early metastasis, resulting in a low survival rate. Histone deacetylase (HDAC) inhibitors showed potential for the treatment of hematological malignancies. In PDAC, the overexpression of HDAC 2 is associated with the epithelial–mesenchymal transition (EMT), principally accompanied by the downregulation of the epithelial marker E-cadherin and increased metastatic capacity. The effector cytokine transforming growth factor-β (TGF β) is known to be a major inducer of the EMT in PDAC, leading to high metastatic and invasive potential. In addition, the overexpression of HDAC 6 in PDAC is associated with reduced apoptosis. Here, we have demonstrated that a novel HDAC 2/6 inhibitor not only significantly increased E-cadherin expression in PANC-1 cells (5.5-fold) and in 3D PDAC co-culture spheroids (2.5-fold) but was also able to reverse the TGF-β-induced downregulation of E-cadherin expression. Moreover, our study indicates that the HDAC inhibitor mediated re-differentiation resulting in a significant inhibition of tumor cell invasion by approximately 60% compared to control. In particular, we have shown that the HDAC inhibitor induces both apoptosis (2-fold) and cell cycle arrest. In conclusion, the HDAC 2/6 inhibitor acts by suppressing invasion via upregulating E-cadherin mediated by HDAC 2 blockade and by inducing cell cycle arrest leading to apoptosis via HDAC 6 inhibition. These results suggest that the HDAC 2/6 inhibitor might represent a novel therapeutic strategy for the treatment of PDAC tumorigenesis and metastasis.

## 1. Introduction

Pancreatic ductal adenocarcinoma (PDAC) is the most common subtype of pancreatic cancer representing one of the most lethal cancers with a 5-year survival rate of less than 11% [1,2]. PDAC displays a large cellular heterogeneity including a highly fibrotic and immunosuppressive tumor microenvironment (TME) [3]. The PDAC tumor microenvironment, which is characterized by the presence of a heterogenous stroma including pancreatic stellate cells (PSCs), tumor endothelial cells, and a variety of immune cells, is mainly responsible for disease progression [3].

During tumorigenesis, pancreatic stellate cells acquire a myofibroblast-like phenotype referred to as cancer-associated fibroblasts (CAFs), which constitute up to 90% of the TME. CAFs excessively secrete extracellular matrix (ECM) proteins, such as collagens, fibronectins, and hyaluronic acid, culminating in the typical desmoplastic environment. Furthermore, the expression of pro-angiogenic factors such as VEGF and PDGF results in the initiation of angiogenesis [4]. Consequently, the creation of atypical multibranched vessels which collapse under the high interstitial pressure of the desmoplastic environment leads to a hypovascular, hypoxic phenotype [5]. Immune cells, including macrophages, T cells, mast cells, and neutrophil granulocytes, infiltrate the desmoplastic stroma and play opposing roles in tumor progression [6]. Inflammatory immune cells, particularly cytotoxic CD8^+^ T cells, foster tumor cell elimination, whereas tumor-associated macrophages and regulatory T cells (Treg) suppress the activation and proliferation of cytotoxic CD8^+^ T cells and of natural killer (NK) cells, thereby contributing to an immunosuppressive tumor microenvironment [7]. Hypoxia promotes the suppression of immune cells by inhibiting the proliferation of anti-tumor immune cells such as cytotoxic T cells or NK cells and increasing the polarization of T cells to Treg cells as well as the polarization of macrophages to an immunosuppressive M2 phenotype [8].

Histone deacetylases (HDACs) are enzymes that deacetylate lysine on histones as well as on many other cellular proteins. Histone deacetylation in general results in the blockage of DNA transcription and the subsequent downregulation of distinct target genes. For example, the HDAC-related suppression of genes encoding for pro-apoptotic proteins like Nur77 or BH3-only protein NOXA leads to an increased cell proliferation [9]. In addition to gene transcription regulation via histone modification, HDACs control many cellular processes including cell cycle progression and cell survival by deacetylating a variety of proteins [10]. As one example, p53 was the first target protein discovered whose deacetylation leads to cell cycle progression and resistance to apoptosis. [11].

To date, the 18 known human histone deacetylases can be divided into four distinct groups. Class I HDACs, which include HDAC 1, 2, 3, and 8, are exclusively located within the nucleus except for HDAC8, while class IIa HDACs (comprising HDAC 4, 5, 7, 9) display nuclear and cytoplasmic localization. HDACs 6 and 10 encompass class IIb HDACs, which are exclusively found in the cytoplasm. In contrast, class IV (currently only consisting of HDAC 11) is predominantly located in the nucleus and subdivided from class I based on the large C-terminus. HDACs require Zn^2+^ for their function. Finally, sirtuins belong to class III HDACs, which require NAD^+^ [12,13].

Aberrant HDACs appear in many different cancer types including PDAC. Recent studies indicate that HDAC class I, HDAC 6, and HDAC 7 tend to be overexpressed in PDAC tissue. Moreover, a high expression of HDAC 1, 7, and 8 is linked to a negative prognosis [14,15]. Different functions of HDACs in PDAC have been reported. For example, class I HDACs promote the epithelial–mesenchymal transition (EMT), leading to a high metastatic capacity, a hallmark in PDAC. Data in mice and in PDAC patients showed a clear correlation between the expression levels of HDAC1 and HDAC2 and the prevalence for metastasis [16]. Furthermore, class IIa HDACs promote angiogenesis by increasing the transcription of hypoxia-inducible factor 1α (HIF-1α) under hypoxic conditions. Moreover, the overexpression of HDAC6 in PDAC results in the upregulation of c-myc which is directly linked to cell proliferation [17]. However, despite the important role of HDAC in cancer progression, further studies are needed to fully understand the involvement of distinct HDACs in PDAC tumorigenesis.

Pancreatic tumor cells undergo the process of the EMT early in their tumorigenic development, transforming from their original epithelial and immobile state to a mesenchymal, fibroblastoid, and mobilized phenotype, an essential step for tumor invasion, dissemination, and metastasis [18]. One key activator of the EMT is the effector cytokine TGF-β which leads to the increased invasion of tumor cells through the downregulation of the epithelial marker E-cadherin [19]. The downregulation of E-cadherin in PDAC is thought to be associated with the increased activity of HDAC 1 and HDAC 2 [20]. Subsequently, low levels of the E-cadherin protein correlate with an invasive and metastatic phenotype [18]. Specifically, a high expression of HDAC 2 is associated with high metastatic capacity resulting from the downregulation of E-cadherin. Moreover, a high expression of HDAC 6 is linked to resistance against apoptosis [14,16].

The latest European Society of Medical Oncology (ESMO) guidelines recommend primary resection followed by adjuvant chemotherapy with either FOLFIRINOX (leucovorin, 5-FU, irinotecan, and oxaliplatin) or, if not eligible for this treatment, gemcitabine/capecitabine for resectable pancreatic cancer as first-line treatment. In metastatic disease, the standard options for first-line chemotherapy are either FOLFIRINOX or gemcitabine/nab (nanoalbumin)-linked paclitaxel [21,22]. However, due to the limited efficacy of the available non-specific cytotoxic drugs and the advanced stage of the disease at the time of diagnosis, with more than 80% of patients already diagnosed with an unresectable or metastatic stage, there is a high unmet medical need for new PDAC treatment strategies [23,24].

HDAC inhibition has lately gained significant attention in cancer drug discovery and development. For instance, the inaugural FDA-approved HDAC inhibitor vorinostat, also known as suberoylanilide hydroxamic acid (SAHA) [19,25], is employed in cutaneous T-cell lymphoma treatment [26]. Although HDAC inhibitors such as vorinostat and panbinostat and class I-specific HDAC entinostat have been investigated for PDAC treatment, they have failed in the latest phase 2 trials due to missing efficacies as monotherapies [20,27]. Additionally, unfavorable side effect profiles of pan-inhibitors arise from HDAC isoenzymes possessing toxic effects on non-tumor cells, mainly through the inhibition of HDAC 1, 2, and 3 leading to the dose limitation of pan-HDAC inhibitors [9].

**Figure 1:**
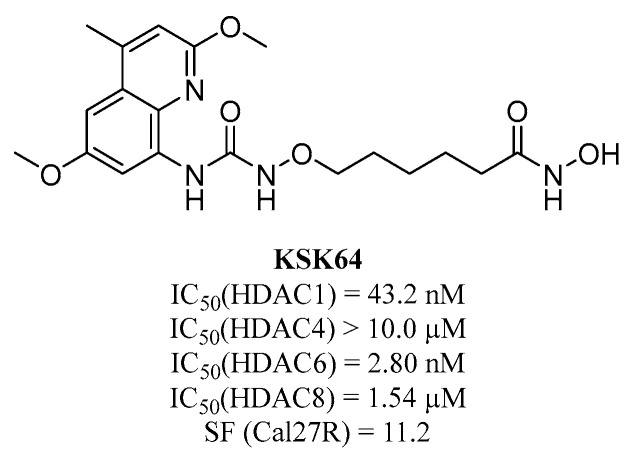
Structure and isozyme profile of KSK64 [28].

In contrast to the first generation pan-HDACi-like panobiostat, **KSK64** (1i) exhibits a refined HDAC isozyme profile with a preference towards class I HDACs (especially HDAC 1, 2) and HDAC6 [28]. This HDAC class I/HDAC6 isozyme profile is presumably the result of the large substituted quinoline Cap group in connection with the novel alkoxyurea moiety (Figure 1).

**Scheme 1:**
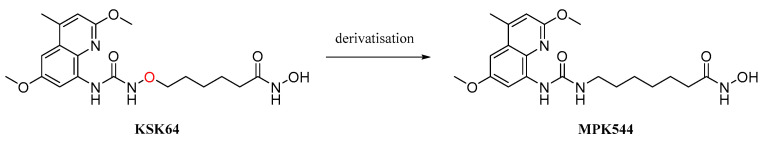
Derivatization of **KSK64**.

However, the N-O bond of the alkoxyurea structure in **KSK64** (1i) could represent a metabolic weak point. Therefore, we decided to synthesize and investigate its carba analogue **MPK544** (Scheme 1). This involved the development of a synthetic strategy for **MPK544** exhibiting a urea connecting unit (CU) and its anticancer property evaluation towards PANC-1 cells.

We here describe the new HDAC inhibitor **MPK544** which was designed based on a pharmacophore model of HDAC class I and IIb inhibitors. The inhibitor contains a substituted quinoline cap group, a urea connecting unit, an alkyl linker, and a hydroxamic acid ZBG. HDAC class I and IIb inhibitors not only target histone proteins within their nuclear complexes by inhibiting HDACs 1–3 but also modulate various cytosolic cancer targets through the inhibition of HDAC 6.

In summary, we demonstrate that our novel HDAC inhibitor **MPK544** exhibits slightly higher cytotoxicity in PANC-1 cells compared to the HDAC 1, 2, 3, and 6 inhibitor vorinostat and comparable efficacy in 3D PDAC spheroid models [29]. Notably, **MPK544** shows a significant reduction in invading a matrigel matrix through the upregulation of the epithelial marker E-cadherin in both PANC-1 cells alone as well as in 3D co-culture spheroids. Overall, our findings provide evidence that HDAC 2/6 inhibitors warrant further investigation as possible novel therapeutic strategies for PDAC treatment.

## 2. Results

**Scheme 2:**
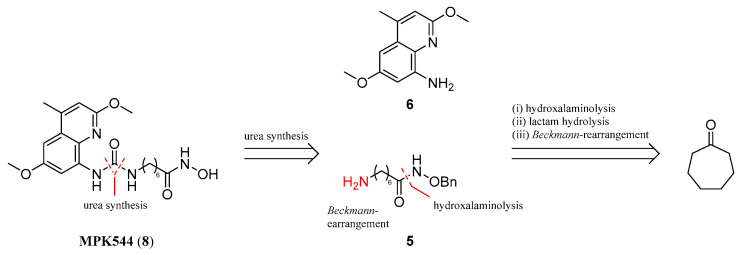
Retrosynthetic analysis of **MPK544**.

In this study, we have synthesized a novel carba analogue of **KSK64** featuring a urea component as the CU, which resulted in the discovery of **MPK544**. The synthetic approach was guided by a detailed retrosynthetic analysis, breaking down the target molecule into readily commercially available synthetic precursors (Scheme 2). The urea derivative was disconnected into the amino-quinoline **7** and the 7-aminoheptanoic alkoxyamide derivative **8**. Our focus was on the efficient synthesis of the latter, which is a key building block for the target molecule. Our retrosynthetic strategy identified a critical path involving the synthesis of 7-amino-*N*-(benzyloxy)heptanamide **5**. This compound can be further derived from cycloheptanone by a Beckmann rearrangement. Our approach provides a reliable route to the alkoxyamide precursor, which is essential for the synthesis of the targeted urea derivative.

The successful implementation of this synthetic pathway, along with a detailed retrosynthetic analysis, underscores the viability of the synthetic method for producing novel **KSK64** derivatives with potent anticancer activity.

**Scheme 3:**
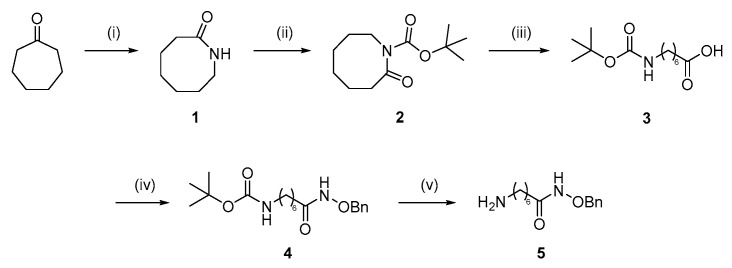
Synthesis of 7-amino-N-(benzyloxy)heptanamide **5**. (i) A total of 1.50 eq hydroxylamine-O-sulfonic acid, HCO_2_H; (ii) 1.00 eq nBuLi, 1.00 eq Boc_2_O, THF; (iii) 10.0 eq LiOH, THF/H_2_O; (iv) 1.00 eq HATU *O*-benzylhydroxylamine, 2.00 eq DIPEA, DMF; (v) 20.0 eq HCl in Dioxane, DCM.

Starting from cycloheptanone, a ring expansion was realized by a Beckmann rearrangement (Scheme 3), followed by an *N*-Boc protection of the resulting lactam **2**. The *N*-Boc-protected lactam **2** was, subsequently, hydrolyzed under basic conditions to obtain the *N*-Boc-protected amino acid **3**. Via a HATU-mediated acylation, **3** was coupled to *O*-benzyl hydroxylamine to obtain **4**. The synthesis of the linker was completed by the Boc deprotection of **4**, yielding **5**.

**Scheme 4:**
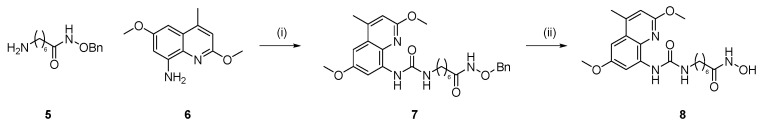
Synthesis of MPK544 (**8**). (i) A total of 0.330 eq triphosgene, 1.00 eq DIPEA, DCM; (ii) H_2_, Pd/C, MeOH.

Afterwards, **5** and the 8-aminoquinoline derivative **6** were used for the synthesis of the urea **7,** in the presence of triphosgene (Scheme 4). Finally, the *O*-benzyl-protected hydroxamic acid precursor **7** was deprotected by catalytic hydrogenation to obtain the hydroxamic acid **MPK544 (8)**.

A cytotoxicity screen of a library of 30 novel HDAC inhibitors against the PDAC cell line PANC-1 revealed **MPK544** as the most potent candidate. The cytotoxic efficacy of **MPK544** was evaluated after 72 h in both primary pancreatic stellate cells (PSCs) and in PANC-1 cells, as well as in 3D co-culture spheroids composed of PANC-1 cells and PSCs. In PANC-1 (IC_50_ 2.95 µM) and PSC cells (IC_50_ 0.68 µM), **MPK544** was more cytotoxic than SAHA (Table 1). In the co-culture spheroids (IC_50_ 0.90 µM), the IC_50_ values of **MPK544** and SAHA were comparable (Table 1). In contrast, the specific HDAC 6 inhibitor tubastatin A did not display any cytotoxicity in these models (Table 1). Noteworthily, **MPK544** was even more cytotoxic in the reported models than the current PDAC standard therapeutic gemcitabine [30]. With respect to enzyme selectivity, **MPK544** showed inhibitory activity against HDAC class I and HDAC 6 (Table 2). In comparison, SAHA was described to inhibit HDAC 1, 2, 3, and 6 [29].

**Figure 2:**
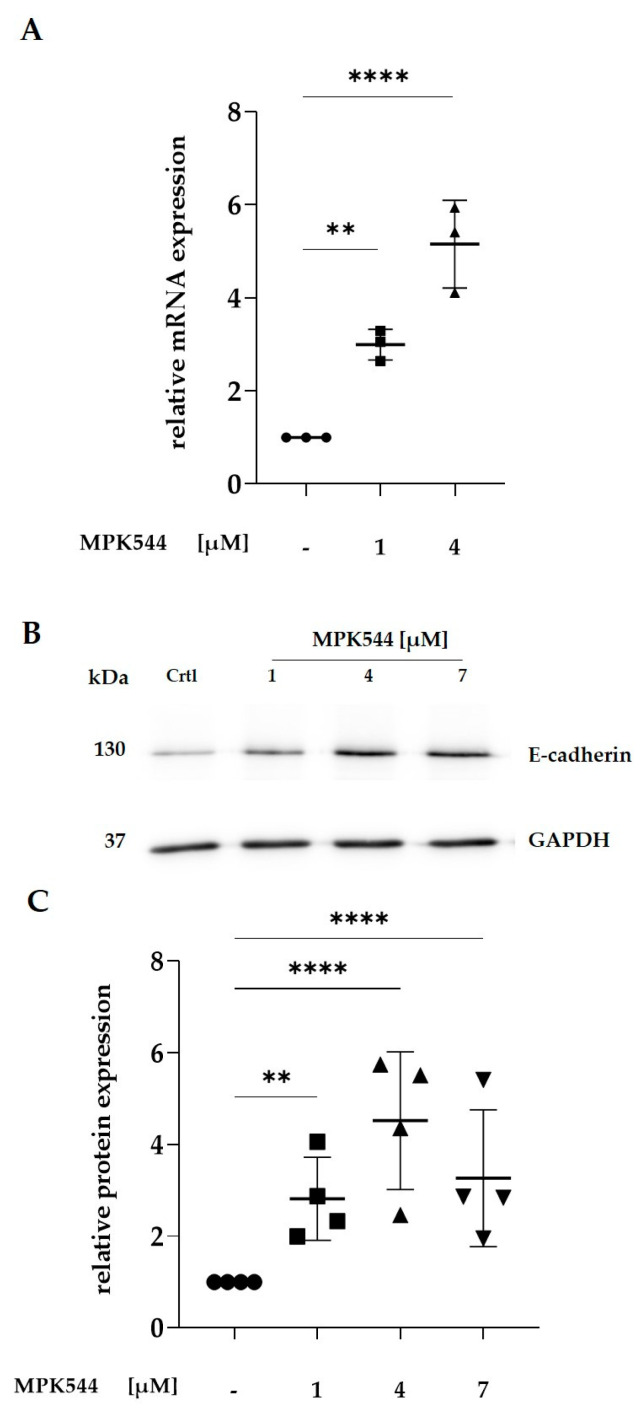
Upregulation of E-cadherin in PANC-1 cells after 24 h. PANC-1 cells were treated with **MPK544**, SAHA, and tubastatin A for 24 h. mRNA levels of E-cadherin in PANC-1 cells were analyzed by qRT-PCR (**A**) and at protein level by Western blot (**B**,**C**) (control ●; 1 µM **MPK544** ▪; 4 µM **MPK544** 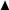; 7 µM **MPK544** 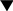). Representative Western blots are shown (**B**). Error bars indicate standard errors of mean (of at least three independent experiments, with; ** = *p* ≤ 0.01; and **** = *p* ≤ 0.0001).

The process of the epithelial–mesenchymal transition (EMT) is a critical step for tumor invasion and metastasis [18]. The characteristic suppression of the epithelial marker protein E-cadherin, controlled through HDAC 2 and HDAC 1 activity, is accompanied with a highly metastatic and invasive phenotype in PDAC [31,32]. The analysis of E-cadherin expression in PANC-1 cells via qPCR (Figure 2A) and Western blot (Figure 2B,C) after treatment with **MPK544**, SAHA, or tubastatin A revealed that **MPK544** increased E-cadherin mRNA levels by 3-fold at a concentration of 1 µM and by 5-fold at 4 µM after 24 h (Figure 2A). After 72 h, compared to SAHA (2-fold), 1 µM **MPK544** induced a 3-fold induction of E-cadherin mRNA levels, whereas treatment with 10 µM tubastatin A did not significantly change E-cadherin mRNA levels in PANC-1 cells (Supplementary Figure S1A). Furthermore, in terms of protein levels, a 5-fold increase in E-cadherin protein expression was observed in the presence of 4 µM **MPK544** after 24 h of incubation (Figure 2B,C). After 72 h treatment with 1 µM **MPK544** (2.2-fold) or SAHA (4-fold) and 4 µM **MPK544** (2.4-fold) or SAHA (6.9-fold), it resulted in a significant protein upregulation of E-cadherin, respectively (Supplementary Figure S1B,C). Again, tubastatin A did not influence E-cadherin protein expression up to a concentration of 10 µM.

**Figure 3:**
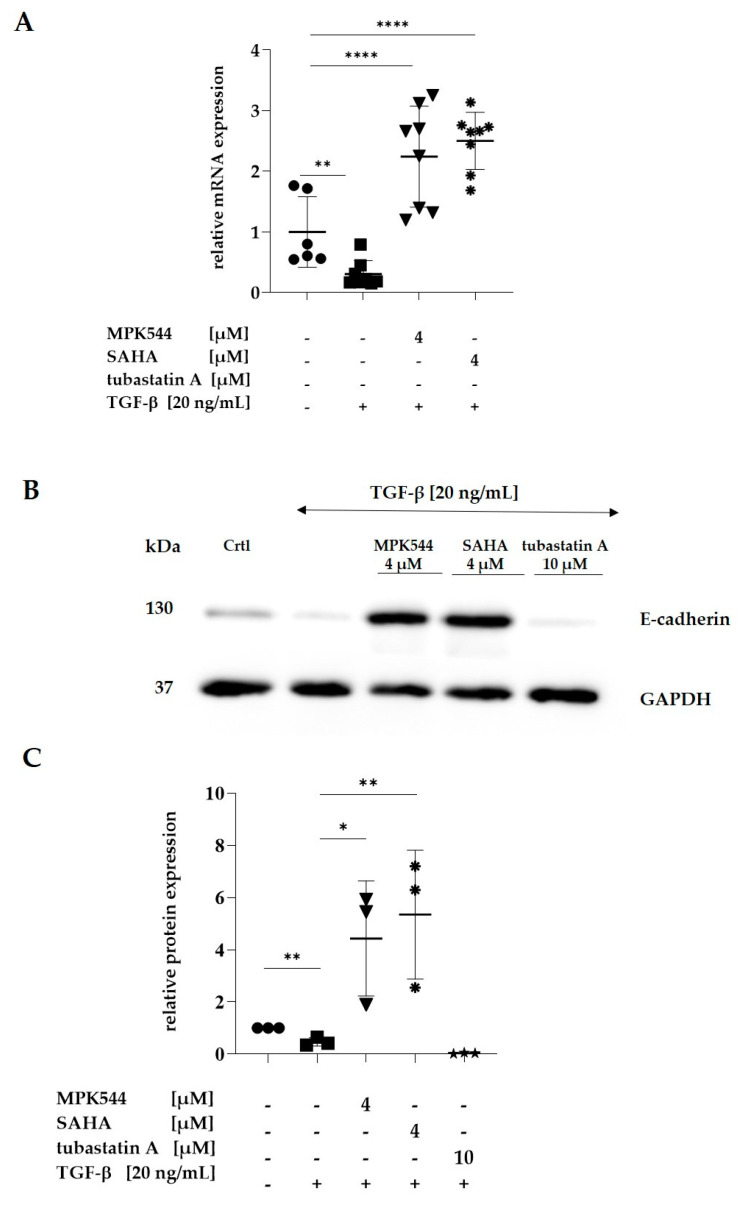
Upregulation of E-cadherin expression in PANC-1 cells. PANC-1 cells were treated with **MPK544**, SAHA, and tubastatin A or with 20 ng/mL TGF-β, for 72 h. mRNA levels of E-cadherin in PANC-1 cells were analyzed by qRT-PCR (**A**) and at protein level by Western blot (**B**,**C**). Representative Western blots are shown (**B**). Control ●; 20 ng/mL TGF-β ▪; 20 ng/mL TGF-β with 4 µM **MPK544** 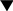; 20 ng/mL TGF-β with 4 µM SAHA 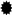; 20 ng/mL TGF-β with 10 µM tubastatin *). Error bars indicate standard errors of mean (of at least three independent experiments, with * = *p* ≤ 0.05; ** = *p* ≤ 0.01; and **** = *p* ≤ 0.0001).

The effector cytokine TGF-β has been identified as a key inducer of the EMT [33]. The TGF-β-induced EMT results in enhanced migratory and invasive properties of PDAC tumor cells, leading to a lower survival rate in patients [34,35,36]. Thus, PANC-1 cells were incubated with TGF-β in combination with **MPK544**, SAHA, or tubastatin A for 72 h, respectively. As expected, TGF-β significantly lowered both E-cadherin mRNA levels as well as E-cadherin protein expression compared to control (Figure 3). Co-treatment with 4 µM **MPK544** resulted in a significant increase in E-cadherin mRNA levels of 7.3-fold (Figure 3A) and of 8.6-fold in the protein level (Figure 3B,C). Co-treatment with 4 µM SAHA resulted in an 8.3-fold upregulation of E-cadherin mRNA levels (Figure 3A), as well as a 10.6-fold increase in the protein level (Figure 3B,C). In line with our previous results, treatment with tubastatin A was not able to reverse the TGF-ß-induced downregulation of E-cadherin (Figure 3A–C).

**Figure 4:**
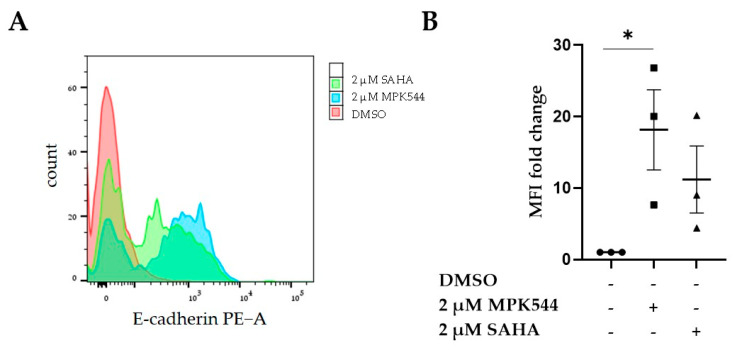
Expression of E-cadherin in 3D co-culture spheroids after treatment with **MPK544** and SAHA. Co-culture spheroids were treated with 2 µM **MPK544** or SAHA for 72 h, respectively. Expression of E-cadherin was quantified via flow cytometry. (**A**) Exemplary overlay histograms for E-cadherin expression after different treatments. (**B**) Quantification of MFI fold change in E-cadherin expression in co-culture spheroids after treatment with 2 µM **MPK544** or SAHA for 72 (control ●; 2 µM **MPK544** ▪; 2 µM SAHA 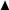). E-cadherin expression was measured in at least 3000 cells. Error bars indicate standard errors of mean (*n* = 3 independent experiments with * = *p* ≤ 0.05.

To additionally analyze E-cadherin protein expression in 3D co-culture spheroids, the spheroids were digested after 72 h of treatment with 2 µM SAHA or **MPK544**. E-cadherin expression was subsequently quantified via flow cytometry. **MPK544** treatment significantly upregulated E-cadherin expression in co-culture spheroids (Figure 4A,B). In co-culture spheroids, **MPK544** treatment increased E-cadherin expression approximately by 20-fold with 2 µM after 72 h. SAHA treatment increased E-cadherin expression in spheroids, with an 11-fold increase with the 2 µM treatment (Figure 4A,B). Additionally, we also confirmed E-cadherin upregulation by the immunofluorescence staining of whole spheroids after treatment with **MPK544** and SAHA compared to the DMSO control (Supplementary Figure S2).

**Figure 5:**
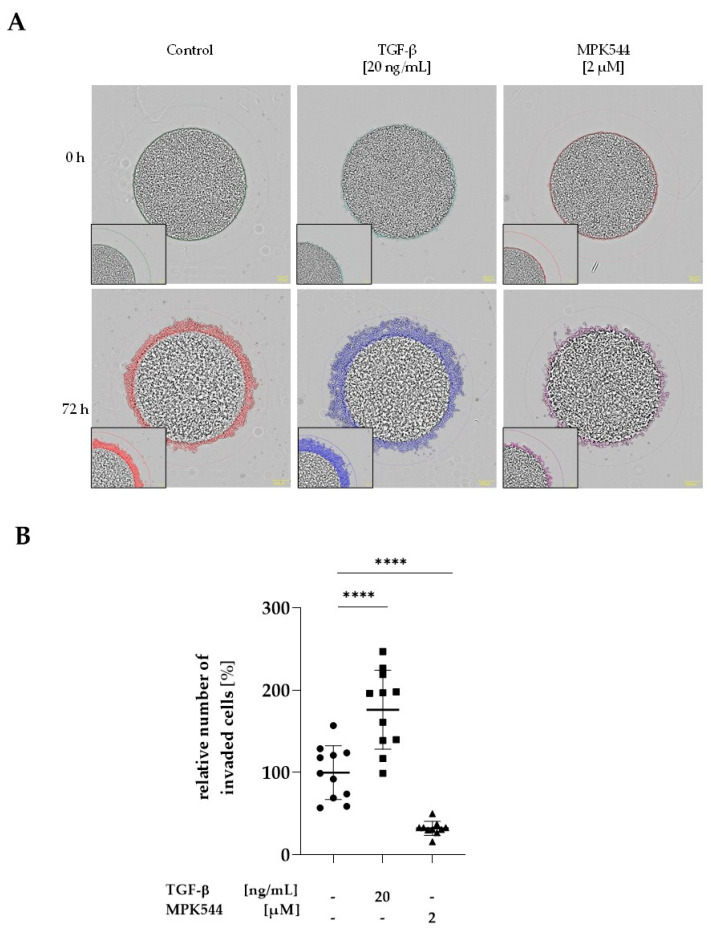
The invasion of PANC-1 cells into a 3D matrix. (**A**) PANC-1 cells were mixed in a ratio of 1:1 with matrigel and seeded into a 24-well plate. After 24 h, a second layer of matrigel was applied, and the cells were incubated for 72 h at 37 °C. The invasion of the PANC-1 cells into the second matrigel layer was studied by phase contrast microscopy (control in red; 20 ng/mL TGF-β in purple; 2 µM **MPK544** in pink). The invasion assay ran for 72 h, and the area of invaded cells into the surrounding matrigel was visualized (control in red; 20 ng/mL TGF-β in purple; 2 µM **MPK544** in pink) and calculated. (**B**) The quantification of the area of invaded cells upon treatment with 2 µM **MPK544** in PANC-1 cells (control ●; 20 ng/mL TGF- β ▪; 2 µM **MPK544** 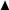). Error bars indicate the standard errors of the mean (*n* = 3 independent experiments with **** = *p* ≤ 0.0001).

In PDAC, HDAC 2 has been shown to suppress the expression of the epithelial marker E-cadherin, thereby promoting the EMT and migration [14,32]. To study the effect of **MPK544** on tumor cell invasion, PANC-1 cells were seeded in a matrigel drop which was surrounded by a second matrigel layer based on a method adapted from Hurley et al. (2017) [37]. The spherical invasion assay enables the chemotaxis-independent observation of cancer cell invasion in contrast to conventional transwell assays. The quantification of the number of cells invading the second matrigel layer revealed that 72 h of treatment with TGF-β increased the number of invading cells by 1.6-fold, while treatment with the subtoxic concentration of 2 µM **MPK544** reduced the number of invading cells by 3-fold compared to control (Figure 5).

**Figure 6:**
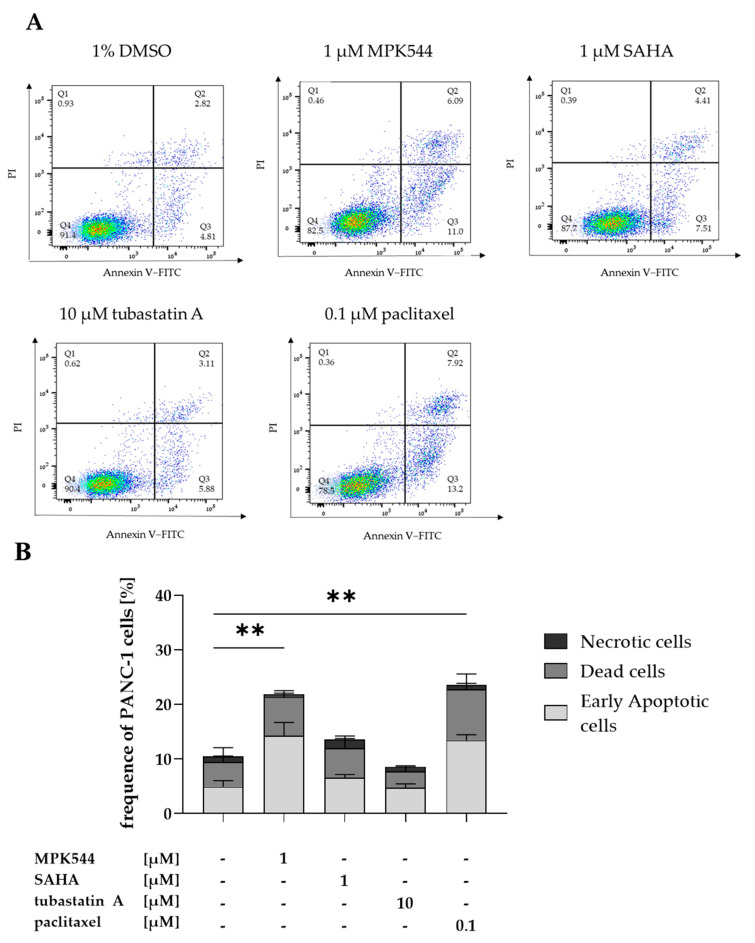
Induction of apoptosis in PANC-1 cells. Cells were treated with 1 µM MKP544, 1 µM SAHA, 10 µM tubastatin A, or 0.1 µM paclitaxel for 48 h. For detection of apoptosis, cells were stained with Annexin V and PI and analyzed by flow cytometry. (**A**) Percentage of apoptotic cells treated with **MPK544**, SAHA, tubastatin A, or paclitaxel are shown in exemplary dot plots. (**B**) Quantification of apoptotic cells after 48 h of treatment. Apoptosis of at least 10,000 cells was analyzed via flow cytometry. Error bars indicate standard errors of mean (*n* = 3 independent experiments with ** = *p* ≤ 0.01).

High expression levels of HDAC 6 have been shown to correlate with resistance to apoptosis [17]. HDAC 2 activity on the other hand regulates cell cycle progression [38]. The commercially available HDAC inhibitors, SAHA and tubastatin A, have been shown to induce apoptosis, as well as cell cycle arrest in the G2/M phase, in different cancer cells, including PANC-1 [39,40,41]. A 48 h treatment with 1 µM **MPK544** significantly increased the amount of early apoptotic and dead cells by 2-fold in comparison to 1% DMSO-treated PANC-1 cells (Figure 6B).

**Figure 7:**
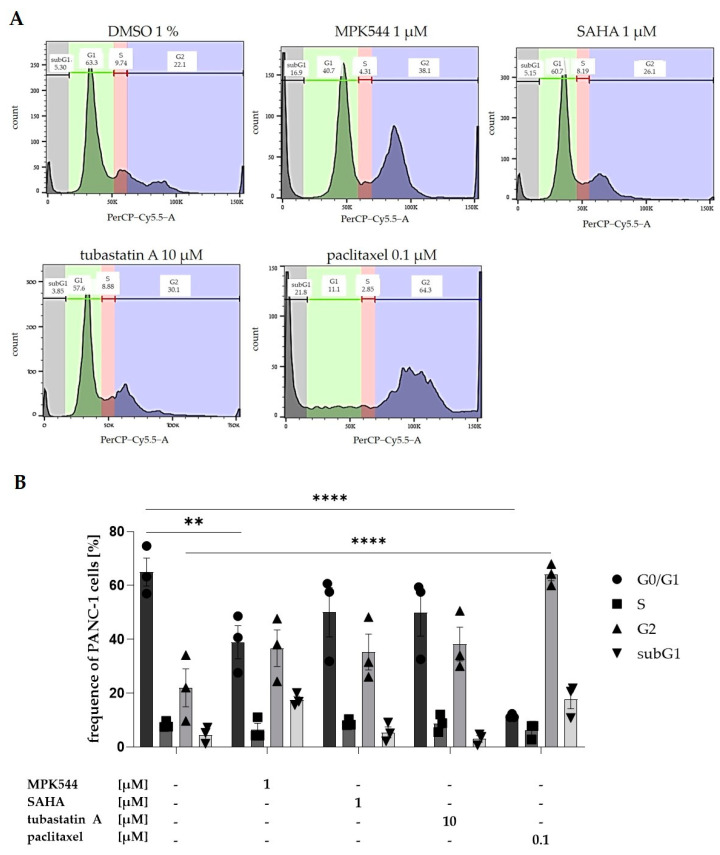
Cell cycle analysis of PANC-1 cells. Cells were treated with 1 µM **MPK544**, 1 µM SAHA, 10 µM tubastatin A, or 0.1 µM paclitaxel for 48 h hours. Cell cycle analysis was performed according to Nicoletti method. (**A**) Percentage of cells in different cell cycles after treatment with **MPK544**, SAHA, tubastatin A, or paclitaxel for 48 h is shown in exemplary histograms. (**B**) Quantification of cell cycle phases. Cell cycles of at least 10,000 cells were analyzed via flow cytometry. Error bars indicate standard errors of mean (*n* = 3 independent experiments with ** = *p* ≤ 0.01; and **** = *p* ≤ 0.0001).

Besides the induction of apoptosis, the influence on the cell cycle of PANC-1 cells after 48 h treatment with 1 µM **MPK544**, 1µM SAHA, 10 µM tubastatin A, or 0.1 µM paclitaxel was analyzed. **MPK544** treatment led to a 1.7-fold reduction in the G1 phase and a 1.6-fold increase in the G2 phase (Figure 7B). Comparable observations were made for the pan-HDAC inhibitor SAHA and the HDAC6 inhibitor tubastatin A, however without achieving significance (Figure 7). Treatment with the tubulin inhibitor paclitaxel induced, as expected, cell cycle arrest in the G2/M phase (Figure 7).

**Figure 8:**
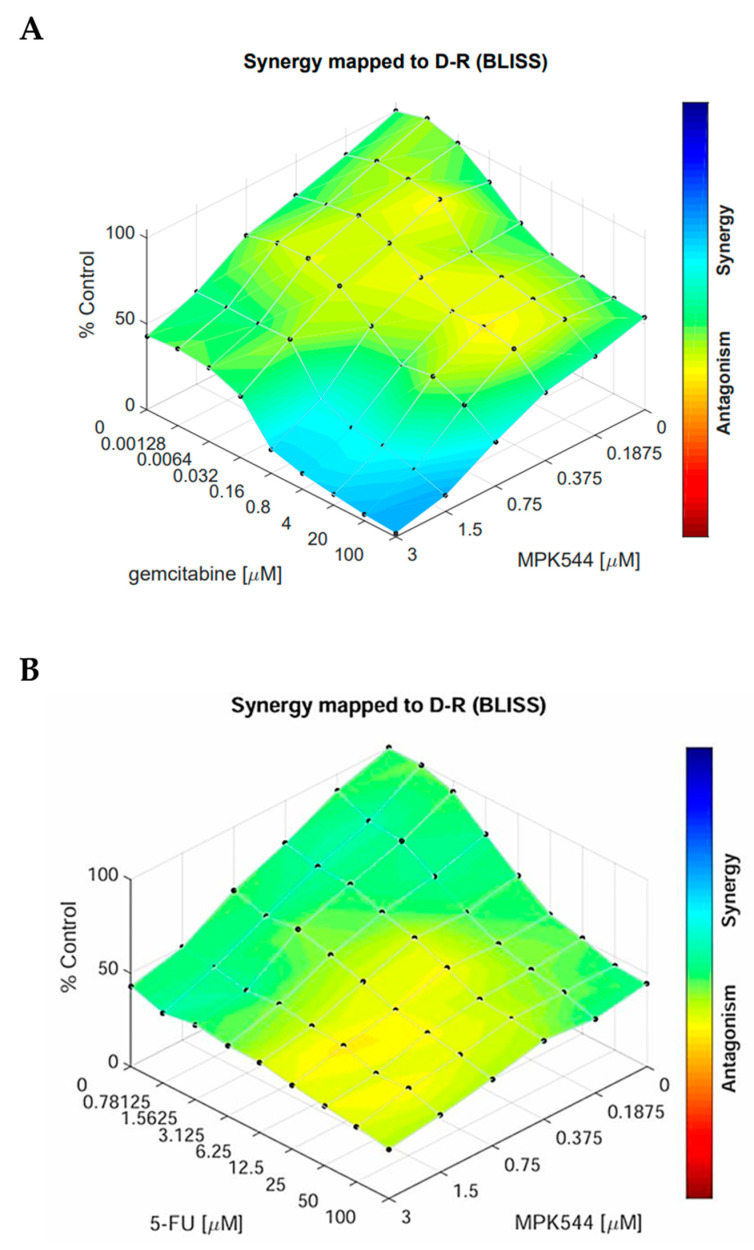
**MPK544** demonstrates a synergistic interaction with the chemotherapeutic drug gemcitabine and no synergistic interaction with 5-fluorouracil (5-FU). A representative Bliss synergy plot of **MPK544** with gemcitabine (**A**) and 5-FU (**B**) in PANC-1 cells (*n* = 3) is depicted. The Bliss synergy was analyzed and visualized with the Combenefit software (version 2.021) [44].

So far, HDACis have failed as monotherapies for solid tumors but have shown the first promising results in combination with chemotherapeutics [42,43]. Therefore, we explored the potential synergism of **MPK544** with the chemotherapeutic drugs gemcitabine and 5-fluorouracil (5-FU) in PANC-1 cells. As shown in Figure 8, we screened **MPK544** against increasing concentrations of gemcitabine or 5-FU, respectively. Indeed, **MPK544** demonstrates a synergistic interaction at concentrations above the IC_50_ value of gemcitabine (0.17 µM) (Figure 8A). However, for 5-FU, no synergistic interaction with **MPK544** could be detected (Figure 8B). Data were generated using the Bliss synergy analysis [44].

## 3. Discussion

HDAC inhibition has gained a lot of attention in the discovery and development of novel anticancer drugs. For example, the HDACi SAHA has been FDA-approved for the treatment of cutaneous T-cell lymphoma [45,46]. In the treatment of PDAC, multiple HDAC inhibitors have demonstrated anti-tumor efficacy in vivo as well as in mouse models but failed in clinical trials [20,42], presumably indicating that HDACis might not be suitable for monotherapy. For PDAC, HDACis are currently investigated in clinical trials as combination therapies with cytotoxic chemotherapeutics such as gemcitabine [43]. We showed that **MPK544** demonstrates a synergistic interaction with gemcitabine in PANC-1 cells. For 5-FU, however, no synergistic interaction with MPK544 was detected. Whether this result can be explained with the selected tumor type or the HDAC inhibitor, selectivity needs to be further explored. In this context, it is worth to mention that a recent study showed a synergistic interaction of 5-FU with the pan-HDACi panobinostat in gastric cancer cell types [47].

Furthermore, we investigated the potential of **MPK544** to reverse the mesenchymal phenotype and reduce invasive properties and cell proliferation in PDAC tumor cells. The cytotoxic effect of **MPK544** was characterized in comparison to SAHA and the specific HDAC 6 inhibitor tubastatin A. In 2D, **MPK544** cytotoxic efficacy was superior to SAHA and tubastatin A. Interestingly, the strongest cytotoxic effect of **MPK544** was observed in co-culture spheroids consisting of PSCs and PANC-1 cells. In PDAC, 90% of the solid tumor is composed of PSCs, generating a desmoplastic microenvironment and leading to an increase in stiffness [3]. As shown by Kim et al. (2018), particularly class I HDACs are overexpressed in CAFs, which explains the remarkably robust cytotoxic efficacy of **MPK544** [48]. This must be taken into account with regard to possible toxic side effects that may occur due to the inhibition of class I HDACs.

To generate specific HDAC inhibitors, it is crucial to understand the critical HDAC targets responsible for the therapeutic effect in a particular cancer type. Studies have shown that class I HDACs and HDAC 6 are the most important targets for cytotoxic anti-cancer effects in PDAC [14,49,50]. Specifically, HDAC 2 is associated with high metastatic capacity resulting from the downregulation of E-cadherin [14]. Thus, HDAC 2 inhibition might be associated with a reversion of the mesenchymal phenotype of PDAC tumor cells into an epithelial phenotype, thereby reducing metastatic capacity and increasing sensitivity to chemotherapeutics [43,51]. We were able to confirm this potential by showing a significant upregulation of E-cadherin at both mRNA and protein levels after treatment with **MPK544**. Furthermore, **MPK544** showed a concentration-dependent effect that was superior to SAHA at a concentration of 1 µM after 24 h. Furthermore, in co-culture spheroids, treatment with **MPK544** led to a significant increase in E-cadherin expression. In contrast, tubastatin A did not induce a significant increase in E-cadherin expression in 2D and 3D models, confirming the hypothesis that HDAC 6 is not involved in the regulation of E-cadherin expression. A key activator of the EMT is the effector cytokine TGF-β which leads to the increased invasion of tumor cells through the downregulation of the epithelial marker E-cadherin [36,52]. Krauß et al. (2022) were able to show that HDAC 2 deactivates the tumor suppressor pathways of TGF-β in PDAC [38]. TGF-β is known to be a potent inducer of the EMT and chemoresistance in PDAC. The most aggressive form of PDAC is classified as a basal-like PDAC which exhibits a TGF-β signature and is enriched in the EMT. Therefore, we investigated whether **MPK544** could reverse the TGF-β-induced EMT. **MPK544** was able to reverse the TGF-β-induced EMT in PANC-1 cells, indicated by the significant upregulation of E-cadherin at the mRNA and protein level. These results are consistent with the findings of Mishra et al. (2017b) showing that the specific class I HDACis domatinostat (4SC-202), entinostat, and mocetinostat were able to reverse the TGF-β-induced EMT [49].

E-cadherin is a calcium-dependent cell adhesion molecule responsible for cell contraction in epithelial tissues. The upregulation of E-cadherin has been shown to be associated with a reduced metastasis rate and invasion of pancreatic cancer cells mediated by the inhibition of HDAC 2. To investigate the invasion capacity, we established a spherical invasion assay adopted from Hurley et al. (2017) [37]. **MPK544** treatment significantly reduced the invasive potential of PANC-1 cells. However, recent studies have revealed that partial EMT (p-EMT) enables the metastasis of circulating tumor cells and is associated with shorter progression-free survival [53,54]. p-EMT may be regulated independently of TGF-β and is therefore fundamentally different from the classical EMT process. In contrast to fully mesenchymal cells, which migrate as individual cells, cells in the p-EMT state migrate in clusters through the vasculature. In future studies, the mechanism by which HDACs regulate p-EMT in PDAC needs to be further investigated.

HDAC inhibition has been shown to affect the cell cycle and induce apoptosis in cancer cells [55]. Our results showed that the inhibition of HDAC 2/6 by **MPK544** resulted in a significant induction of apoptosis and cell cycle shift in the G2/M phase. In agreement with the results of Wang et al. (2012), treatment with the HDAC 6-specific inhibitor tubastatin A showed minimal effects on the cell cycle and the induction of apoptosis [50]. These results support the findings of Krauß et al. (2022) who showed that the depletion of HDAC 2 led to cell cycle arrest at the G2/M phase in murine PDAC [38]. HDAC 6 is a unique member of the HDAC family due to its functionality and localization. It is primarily localized in the cytoplasm and has two catalytic deacetylase domains [56]. In addition, HDAC 6 has been reported to interact with a wide range of different proteins such as α-tubulin, heat shock protein 90 (Hsp90), ERK1, and many others [57,58]. Through these interactions, HDAC 6 regulates various cellular processes such as proliferation and apoptosis [59]. However, studies have shown that targeting HDAC 6 alone has not led to any significant benefit in solid tumors. Interestingly, several studies have shown that HDAC 6 inhibitors in combination therapy with chemotherapeutics increased the cytotoxic efficacy of the latter [60].

In conclusion, **MPK544** acts by suppressing invasion via upregulating E-cadherin mediated by HDAC 2 blockade and by inducing cell cycle arrest leading to apoptosis via HDAC 6 inhibition. Furthermore, a synergistic interaction of MPK544 with the chemotherapeutic agent gemcitabine could be demonstrated. Overall, these results suggest that HDAC 2/6 inhibitors might represent a novel therapeutic strategy for the treatment for PDAC.

## 4. Materials and Methods

### 4.1. Cell Culture

The PANC-1 cell line, acquired from the American Type Culture Collection (ATCC), was cultured in Dulbecco’s Modified Eagle’s Medium (DMEM) (#41965039, Gibco, Grand Island, NY, USA) supplemented with 10% fetal bovine serum (FBS) (#10270-106, Gibco), as well as 1% penicillin–streptomycin (#15140122, Gibco, Grand Island, NY, USA). Pancreatic stellate cells (PSCs) were provided by Dr. Erkan of Koç University Hospital in Turkey. The ethics committee for the biomedical sciences at KOÇ University granted ethical approval, and all patients consented in writing. Pancreatic stellate cells were obtained from patients diagnosed with ductal adenocarcinoma and cultured in DMEM/F12 (#11320074, Gibco, Grand Island, NY, USA) with 20% fetal bovine serum (FBS) and 1% pen-strep under aseptic conditions. Both cell types were incubated at 37 °C in a humidified atmosphere supplemented with 5% CO_2_.

### 4.2. Spheroid Formation

PANC-1 spheroids were formed by adding 5000 PANC-1 cells per well into an ultra-low attachment (ULA)-coated surface plate (#650970, Greiner Bio-One, Kremsmünster, Austria) in DMEM. Co-culture spheroids were generated according to our previous study [30]. The compound treatment of PANC-1 spheroids and co-culture spheroids was initiated on day 4 following cell seeding.

### 4.3. Cell Viability Assays

Cell viability in both PANC-1 and PSC cells was assessed using the PrestoBlue HS Cell Viability Assay (#P50201, Thermo Scientific, Waltham, MA, USA) in 96-well plates (#655090, Greiner Bio-One, Kremsmünster, Austria), seeding 5000 cells per well for PANC-1 and 2000 cells per well for PSCs. Compounds were incubated for 72 h. Fluorescence values were recorded using the Tecan SPARK instrument (Tecan Group, Männedorf, Switzerland). The cell viability of 3D spheroids was analyzed with the CellTiter-Glo^®^ 3D Cell Viability Assay (#G9681, Promega, Madison, WI, USA) according to the manufacturer’s protocol. Luminescence values were measured with the Tecan SPARK instrument (Tecan Group, Männedorf, Switzerland).

### 4.4. Western Blot Analysis

PANC-1 was seeded onto 6-well plates (#657180, Greiner Bio-One, Kremsmünster, Austria) containing 1.8 mL DMEM supplemented with 10% FBS and 1% pen-strep. Cells were treated either with **MPK544**, SAHA, or tubastatin A for 72 h. Cells were lysed with 500 µL of radioimmunoprecipitation assay (RIPA) buffer, along with a protease inhibitor (#11836170001, Roche, Basel, Switzerland) and phosphatase inhibitor (#04906845001, Roche). The BCA protein assay kit (#23227, Thermo Scientific, Waltham, MA, USA) was used to determine protein concentration. After denaturation at 95 °C for 10 min, the proteins were loaded onto a 10% SDS gel and separated using the Biometra Eco-Mini Buffer Tank system (Analytik Jena, Jena, Germany, #846-017-103/017-170). Protein transfer onto a PVDF membrane (BioRad Laboratories, Inc., Hercules, CA, USA, #1620177) was facilitated using the Biometra Fastblot system (#846-015-299 Analytik Jena, Jena, Germany). The membrane was incubated overnight at 4 °C with the following antibodies: E-cadherin (BD Bioscience, Heidelberg, Germany, #610182) and GAPDH (Cell signaling, Danvers, MA, USA, #2118). After three washes with 1x TBS-T, the corresponding secondary antibody labeled with horseradish peroxidase was incubated at room temperature for one hour (Goat anti-rabbit, #31460, Goat anti-mouse #31432, Thermo Fisher Scientific, Waltham, MA, USA). Antibody binding was detected using the SuperSignal West Pico Plus substrate kit (#34577, Thermo Fisher Scientific, Waltham, MA, USA) and visualized using the ChemStudio 159 imager (#849-97-0928-04, Analytik Jena, Jena, Germany).

### 4.5. Quantitative Real-Time PCR (qRT-PCR)

To determine E-cadherin mRNA levels after treatment with MKP544, the following primers from Eurofins were utilized (Ebersberg, Germany): E-cadherin (forward, 5′ CGAGAGCTACACGTTCACGG-3′, reverse 5′-GGGTGTCGAGGGAAAAATAGG-3′) (Anzai et al. 2017) and GAPDH (forward, 5′-TGCACCACCAACTGCTTAGC-3′; reverse, 5′-GGCATGGACTGTGGTCATGAG-3′). PANC-1 cells were incubated with **MPK544**, SAHA, or tubastatin A for 24 or 72 h. The total RNA was extracted using the RNeasy MiniKit (#74104, Qiagen, Hilden, Germany) as described in the manufacturer’s instructions. The determination of the RNA concentration was performed using the Tecan Spark NanoQuant plate (Tecan Group, Männedorf, Switzerland). The cDNA synthesis was conducted with 1 µg of isolated mRNA using the QuantiTect Reverse Transcription Kit (#205311, Qiagen, Hilden, Germany). The qRT-PCR was carried out with 50 ng cDNA using the Luna^®^ Universal qPCR Master Mix (# M3003L, New England BioLabs^®^ Inc., Ipswich, MA, USA). Ct values were calculated with the ΔΔCT method. Sample values were normalized to the housekeeping gene GAPDH (glyceraldehyde 3-phosphate dehydrogenase). Analytical evaluation was performed with the qPCRsoft 4.1 software (Analytic Jena, Jena, Germany).

### 4.6. Spherical Invasion Assay

The invasion assay was adapted from Hurley et al. (2017) [37]. In summary, PANC-1 cells were diluted 1:1 with matrigel (#356230, Corning, Corning, NY, USA). Compounds were applied to the cell suspension in a 24-well plate. The matrigel was allowed to polymerize for 30 min at 37 °C. After a 24 h incubation period, a second matrigel layer was added on top of the first. The invasion of the PANC-1 cells into the second layer of matrigel was studied for 72 h with the CQ1 Confocal Imaging Cytometer (#90ZA00673, Yokogawa, Tokyo, Japan). The invaded number of cells was quantified at 0 h and after 72 h with the CellPathFinder software (version 3.06.06.06, Yokogawa). Machine learning was used to automatically identify and count each invasive cell in the brightfield mode. To capture all cells invaded in the second matrigel layer, the analysis radius was set to 1500 µm.

### 4.7. Immunofluorescence of Whole Spheroids

Spheroids were cultivated for 4 days, followed by 72 h of treatment. The protocol was adapted from Xie et al. (2020), and the spheroids were fixed with 4% paraformaldehyde for 1 h at room temperature [30]. The primary antibody staining process involved permeabilization using 0.25% Triton X-100, followed by blocking with a solution of 0.1% BSA and 0.01% Tween 20 in PBS. Spheroids were incubated with 100 µL of primary antibody solution (Human Ki67/MKI67 (#MAB7617, R&DSystems, Minneapolis, MN, USA), E-cadherin (#610182, BD Biosciences, Franklin Lakes, NJ, USA) for 24 h at 4 °C. The secondary antibody solution (Alexa Flour 647 donkey (H+L) anti-mouse (#A-31571, Invitrogen, Waltham, MA, USA), Alexa Flour 488 donkey (H+L) anti-rabbit (#A-21206, Invitrogen)) including 2 µg/mL DAPI (#D9542, Sigma-Aldrich, Saint Louis, MI, USA) was incubated at 4 °C for 24 h. The clearing process was performed by sequential incubation in six ethanol solutions with an increasing concentration (30%, 50%, 70%, 90%, 96%, and 100%), each for 30 min. A final clearing step involved a benzyl alcohol/benzyl benzoate solution with a 1:2 ratio for 1 h. Afterwards, the spheroids were transferred to an imaging plate (# 89626, ibidi GmbH, Gräfelfing, Germany) and imaged with the CQ1 Confocal Imaging Cytometer (Part Number 90ZA00673, Yokogawa). Z-stacks were obtained with 100 stacks per spheroid (1 slice/µm) and analyzed using the CellPathFinder software.

### 4.8. Flow Cytometry

Co-culture spheroids were generated as described above. Then, 4 days after seeding, spheroids were treated with drug candidates and harvested after 72 h of incubation. After washing with PBS, spheroids were digested into single cell suspension using 500 µL Accumax Cell Dissociation Solution (#ACM-1F, Capricorn Scientific GmbH, Ebsdorfergrund, Germany). The single cell suspensions were transferred onto a 96 V bottom plate (#83.3926, Sarstedt, Nümbrecht, Germany) and stained in Automacs Running Buffer with REA Control (S)-PE (#130-113-438, Miltenyi, Bergisch Gladbach, Germany), E-cadherin-PE (#130-111-992, Miltenyi) antibodies according to manufacture introductions for 10 min at 4 °C. For the analysis of apoptosis and the cell cycle, PANC-1 cells were cultured in 6-well plates. After treatment for 48 h, cells were harvested and washed with PBS. Staining was performed using the Annexin V-FITC/propidium iodide (PI) Apoptosis Detection Kit from Elabscience (#E-CK-A211, Housten, TX, USA) according to the manufacturer’s instructions. For cell cycle analysis, cells were washed with PBS and suspended in 100 µL of hypotonic buffer (0.1% Triton X-100, 1% sodium citrate, and 0.05 mg/mL PI in double-distilled water). The cells were incubated for 10 min at room temperature. Flow cytometry was performed with the BD FACSLyric flow cytometer (#87135, BD Bioscience, Franklin Lakes, NJ, USA) and analyzed with the FlowJo software (version 10.8.1).

### 4.9. Combinatorial Drug Testing

PANC-1 cells were seeded onto 96-well plates (#655090, Greiner Bio-One, Kremsmünster, Austria). Then, 24 h after seeding, the cells were treated with increasing concentrations in 9 × 6 dose–response matrices. For MPK544, the concentration range was 0.1875 µM up to 3 µM, for gemcitabine from 1.28 nM up to 100 μM, and for 5-fluorouracil (5-FU) from 0.78125 µM up to 100 µM, treated at 1:2, 1:5, and 1:2 dilutions, respectively. Cell viability was monitored after 72 h using the PrestoBlue HS Cell Viability Assay (as described in 4.3.). Bliss synergy scores were determined using the Combenefit synergy analysis software (version 2.021) [44].

### 4.10. Statistical Analysis

GraphPad Prism 8.4.3 software (GraphPad Software, Boston, MA, USA) was utilized for statistical and graphical analysis. The IC50 values were acquired for assessing cell viability through non-linear regression. A one-way analysis of variance (ANOVA, New Providence, NJ, USA) and unpaired *t*-test were employed to scrutinize statistical data, with *p*-values < 0.05 considered statistically significant.

## Figures and Tables

**Table 1:** The cytotoxic efficacy of HDAC inhibitors represented by the respective IC50 values [µM] in 2D PANC-1 and pancreatic stellate cell (PSC) cultures as well as in 3D co-culture spheroids after 72 h incubation. The arithmetic means and their respective standard deviations of three independent experiments are depicted.

HDAC Inhibitors	PANC-1	PSC	Co-Culture Spheroid
	** IC_50_ in µM **		
** MPK544 **	2.95 ± 0.45	0.68 ± 0.48	0.90 ± 0.15
** KSK64 **	7.04 ± 0.58	14.87 ± 4.55	3.30 ± 0.58
SAHA	3.65 ± 0.43	3.50 ± 0.99	0.85 ± 0.25
tubastatin A	>100	>100	>100

**Table 2:** HDAC 2, HDAC 4, and HDAC 6 inhibition assay of **MPK544**.

HDAC 2	HDAC 4	HDAC 6
IC_50_ in µM		
0.058 ± 0.02	>100	0.097 ± 0.062

## Data Availability

Data is contained within the article and Supplementary Materials.

## Supplementary Materials

The following supporting information can be downloaded at https://www.mdpi.com/article/10.3390/ph17060752/s1. References [61,62,63] are cited in Supplementary Material.
