# Supplementary material for: Novel Histone Deacetylase (HDAC) Inhibitor Induces Apoptosis and Suppresses Invasion via E-Cadherin Upregulation in Pancreatic Ductal Adenocarcinoma (PDAC)
Source: Pharmaceuticals (Basel). 2024 Jun 7;17(6):752. doi: 10.3390/ph17060752 (PMC11206922; doi:10.3390/ph17060752)
Supplement: Supplementary file 1 [file pharmaceuticals-17-00752-s001.zip › pharmaceuticals-2953735-supplementary.pdf]

1. General Information

1.1. Reaction, monitoring and purification

Chemicals and solvents were purchased from commercial suppliers (Sigma-Aldrich, Acros Organics, TCI, Fluorochem ABCR, Alfa Aesar, J&K, Carbolution) and used without further purification. Dry solvents were obtained from Acros Organics. Ambient or room temperature correspond to 22°C. The reaction progression was monitored using Thin-Layer-Chromatography plates by Macherey Nagel (ALUGRAM Xtra SIL G/UV_{254}). Visualisation was achieved with ultraviolet irradiation (254 nm) or by staining with a $KmnO_4$-solution (9 g $KmnO_4$, 60 g K_2CO_3, 15 mL of a 5% aqueous NaOH-solution, ad 900 mL demineralised water). Purification was either performed with prepacked Silica cartridges (RediSep® Rf Normal Phase Silica, RediSep® Rf RP C18) for flash column chromatography (CombiFlashRf200, TeleDyneIsco) or by recrystallisation. Different eluent mixtures of solvents (hexane and ethyl acetate or dichloromethane and methanol) served as the mobile phase for flash column chromatography and are stated in the experimental procedure.

1.2. Analytics

An NMR-Spectrometer by Bruker (Bruker Avance III – 300, Bruker Avance DRX – 500 or Bruker Avance III – 600) were used to perform ^{1}H- and^{13}C-NMR experiments. Chemical shifts are given in parts per million (ppm), relative to residual non-deuterated solvent peak (^{1}H-NMR: DMSO-d_6 (2.50), ^{13}C-NMR: DMSOd_6 (39.52). Signal patterns are indicated as: singlet (s), doublet (d), triplet (t), quartet (q), or multiplet (m). Coupling constants, J, are quoted to the nearest 0.1 Hz and are presented as observed. ESI-MS was carried out using Bruker Daltonics UHR-QTOF maXis 4G (Bruker Daltonics) under electrospray ionization (ESI). The above-mentioned characterisations were carried out by the HHU Center of Molecular and Structural Analytics at Heinrich-Heine University Düsseldorf (http://www.chemie.hhu.de/en/analytics-center-hhucemsa.html). APCI-MS was carried out with an Advion expression^L CMS. Melting points were determined using a Büchi M-565 melting point apparatus (uncorrected). Analytical HPLC was carried out on a Knauer HPLC system comprising of an Azura P6.1L pump, an Optimas 800 autosampler, a Fast Scanning Spectro-Photometer K-2600 and a Knauer Reversed Phase column (SN: FK36). Evaluated compounds were detected at 254 nm. The solvent gradient table is shown in **Error! Reference source not found.**. The purity of all final compounds was 95% or higher.

Supplementary Table S1. The solvent gradient table for analytic HPLC analysis.

Time / min	Water + 0.1% TFA	ACN + 0.1% TFA
Initial	90	10
0.50	90	10
20.0	0	100
30.0	0	100

31.0	90	10
40.0	90	10

2. Synthetic procedures

*2.1. Synthesis of azocan-2-one (**1**)*

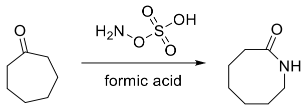

1 was accessed via a Beckmann rearrangement. [1]

All spectroscopic data were in agreement with literature. [1]

*2.2 Synthesis of tert-butyl 2-oxoazocane-1-carboxylate (**2**)*

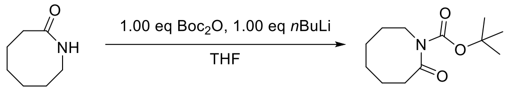

2 was synthesised according to Giovannini *et. Al.*. [2]

All spectroscopic data were in agreement with literature. [2]

*2.3 Synthesis of 7-((tert-butoxycarbonyl)amino)heptanoic acid (**3**)*

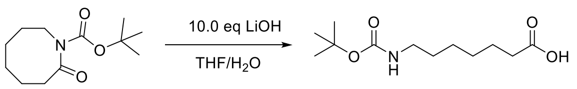

1.00 eq (4.40 mmol, 1.0 g) of **2** was dissolved in 22 mL of THF. 10.0 eq (44.0 mmol, 1.05 g) of LiOH was dissolved in 4 mL of dH_2O and added to the reaction. After 16 h at ambient temperature, the reaction solution was poured on 100 mL of H_2O, acidified with 1 M $HCl_{(aq)}$ and the product extracted with EtOAc. The combined organic layers were dried over Na_2SO_4 and the solvent removed under reduced pressure. 0.97 g (3.95 mmol, 90 %) of **3** was obtained as a white solid.

^{1}H NMR (600 MHz, DMSO-d_6) δ 11.96 (s, 1H), 6.74 (t, *J* = 5.8 Hz, 1H), 2.94 – 2.82 (m, 2H), 2.18 (t, *J* = 7.4 Hz, 2H), 1.46 (td, *J* = 14.6, 7.3 Hz, 2H), 1.36 (s, 10H), 1.23 (tq, *J* = 8.7, 5.2, 3.8 Hz, 4H).

^{13}C NMR (151 MHz, DMSO-d_6) δ 174.5, 155.6, 77.3, 40.1, 33.6, 29.4, 28.3, 28.3, 26.0, 24.5.

M.p.: 47.5 °C; **MS** (+APCI): 190 $[M-{}^{t}BU+2H]^+$.

*2.4 Synthesis of tert-butyl (7-((benzyloxy)amino)-7-oxoheptyl)carbamate (**4**)*

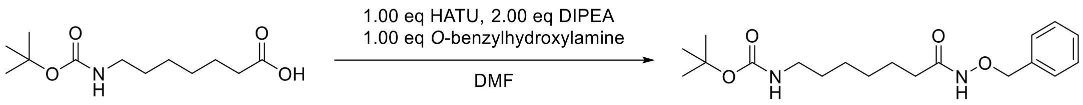

1.00 eq (3.39 mmol, 831 mg) of **6**, 1.00 eq (3.39 mmol, 1.29 g) of HATU and 2.00 eq (6.77 mmol, 880 mg, 1.19 mL) of DIPEA were combined in 10 mL of DMF. After 10 minutes at RT, 1.00 eq (3.39 mmol, 417 mg) of *O*-benzyl hydroxalamine were added to the reaction mixture and stirred for 16 h at ambient temperature. Subsequently, the solvent was removed *in vacuo* and the residue resuspended in 50 mL of EtOAc. The organic layer was washed with 10 % aq. Citric acid (x3), aq. Sat. $NaHCO_3$ (x3), brine (x1) and dried over Na_2SO_4. 1.12 g (3.20 mmol, 94 %) of **4** was obtained as a white solid after flash chromatography (*n*-hexane/EtOAc).

^{1}H NMR (600 MHz, DMSO-d_6) δ 10.9 (s, 1H), 7.7 – 7.2 (m, 5H), 6.8 (t, *J* = 5.8 Hz, 1H), 4.8 (s, 2H), 2.9 (q, *J* = 6.7 Hz, 2H), 1.9 (t, *J* = 7.3 Hz, 2H), 1.5 – 1.4 (m, 2H), 1.4 (s, 12H), 1.2 (p, *J* = 3.9, 3.4 Hz, 4H).

^{13}C NMR (75 MHz, DMSO-d_6) δ 169.3, 155.5, 136.0, 128.6, 128.1, 128.1, 77.2, 76.7, 39.7, 32.1, 29.3, 28.2, 28.1, 25.9, 24.8.

M.p.: 56.1 °C; **MS** (+APCI): 295 [M-^{t}BU+2H]$^+$.

*2.5 Synthesis of 7-amino-N-(benzyloxy)heptanamide hydrochloride (**5**)*

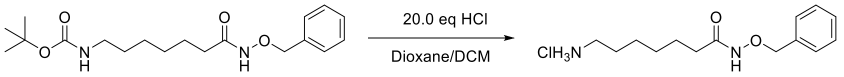

1.00 eq (2.74 mmol, 960 mg) of **4** was dissolved in 27.4 mL of DCM and cooled on ice. Subsequently, 20 eq (54.8 mmol, 13,7 mL) of a 4 M HCl in dioxane solution were added and stirred for 4 h on ice. The product was precipitated by the addition of 1 volume of hexane and the product collected by filtration. 760 mg (2.65 mmol, 97 %) of **5** was obtained as a white solid.

^{1}H NMR (600 MHz, DMSO-d_6) δ 11.12 (s, 1H), 8.10 (s, 3H), 7.44 – 7.28 (m, 5H), 4.78 (s, 2H), 2.71 (qt, *J* = 9.4, 4.6 Hz, 2H), 1.96 (t, *J* = 7.3 Hz, 2H), 1.54 (p, *J* = 7.6 Hz, 2H), 1.48 (p, *J* = 7.4 Hz, 2H), 1.33 – 1.25 (m, 2H), 1.25 – 1.12 (m, 2H).

^{13}C NMR (75 MHz, DMSO-d_6) δ 169.2, 136.0, 128.6, 128.2, 128.1, 76.7, 38.6, 32.0, 27.8, 26.6, 25.4, 24.6.

M.p.: 110.3°C; **MS** (+APCI): 287 [M+H]$^+$.

*2.6 Synthesis of 2,6-dimethoxy-4-methylquinolin-8-amine (**6**)*

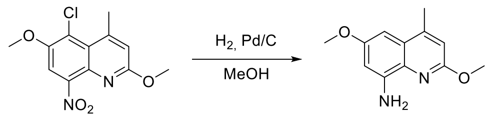

1.00 eq (3.54 mmol, 1 g) of 5-chloro-2,6-dimethoxy-4-methyl-8-nitroquinoline [3]was dissolved in 70 mL of Methanol. After the addition of 5 mol% of 10 % Pd/C (0.177, 0.188 g) the catalytic hydrogenation was performed for 5 h. Subsequently, 10.0 eq of TEA (35.4 mmol, 3.58 g, 2.6 mL) were added to the reaction mixture. The solution was filtered over celite®, the solvent removed under reduced pressure and the product purified by flash column chromatography (Hex/DCM + 0.1 % TEA). 461 mg (2.11 mmol, 60 %) of **6** was obtained as a white solid.

^{1}H NMR (600 MHz, DMSO-d_6) δ 6.79 (t, *J* = 1.2 Hz, 1H), 6.50 (dd, *J* = 2.6, 1.0 Hz, 1H), 6.45 (dd, *J* = 2.6, 1.0 Hz, 1H), 5.69 (s, 2H), 3.93 (d, *J* = 1.0 Hz, 3H), 3.79 (d, *J* = 1.1 Hz, 3H), 2.49 (t, *J* = 1.1 Hz, 3H).

^{13}C NMR (151 MHz, DMSO-d_6) δ 158.3, 156.5, 146.3, 145.0, 130.6, 125.6, 112.3, 99.9, 90.8, 54.9, 52.5, 18.7.

M.p.: 135.7 °C; **HPLC**: R_t= min, purity %, **MS** (+APCI): 219 $[M+H]^+$.

*2.7. Synthesis of N-(benzyloxy)-7-(3-(2,6-dimethoxy-4-methylquinolin-8-yl)ureido)heptanamide (**7**)*

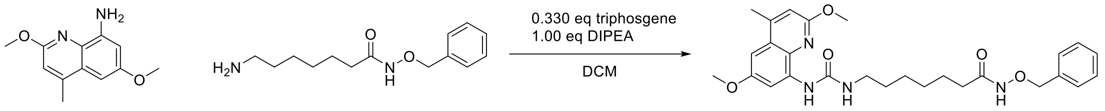

0.330 eq (0.660 mmol, 196 mg) of triphosgene was dissolved in 30 mL of dry DCM and cooled in ice. 1.00 eq of **5** was dissolved in 10 mL of dry DCM and added dropwise to the triphosgene solution over a period of 30 minutes. 1.0 eq of DIPEA was mixed with 10 mL of dry DCM and added dropwise to the reaction solution over a period of 30 minutes and stirred for an addition 30 minutes at RT. Subsequently, 1.00 eq of the respective amine derivative **8** was dissolved in 10 mL of dry DCM and added over a period of 30 minutes. The reaction was stirred for 16 h at ambient temoerature, the solvent was removed *in vacuo* and the residue resuspended in EtOAc. Afterwards, the organic phase was washed with dH_2O (x3), brine (x1) and dried over Na_2SO_4. Finally, the product was purified by flash column chromatography (Hexane/DCM).

7 was synthesised on a 1.83 mmol scale with a yield of 35 % (315 mg, 0.634 mmol) and obtained as a white solid.

^{1}**H NMR** (300 MHz, DMSO-*d_6*) δ 8.67 (s, 1H), 7.50 (t, J = 5.5 Hz, 1H), 7.43 – 7.28 (m, 5H), 6.89 (d, J = 1.1 Hz, 1H), 6.79 (d, J = 2.7 Hz, 1H), 4.78 (s, 2H), 4.05 (s, 3H), 3.84 (s, 3H), 3.13 (q, J = 6.6 Hz, 2H), 2.55 (d, J = 1.0 Hz, 3H), 1.96 (t, J = 7.2 Hz, 2H), 1.49 (dp, J = 13.7, 7.3 Hz, 5H), 1.29 (h, J = 5.3 Hz, 5H).

^{13}**C NMR** (75 MHz, DMSO- *d_6*) δ 169.3, 159.0, 155.8, 154.8, 146.7, 136.1, 130.6, 128.6, 128.1, 128.0, 124.8, 112.6, 105.8, 95.2, 76.7, 55.1, 53.1, 32.1, 29.5, 28.2, 26.1, 24.8, 18.4.

M.p.: 171.2°C; **MS** (+APCI): 496 $[M+H]^+$.

*2.8.1 Synthesis of 7-(3-(2,6-dimethoxy-4-methylquinolin-8-yl)ureido)-N-hydroxyheptanamide (**8**)*

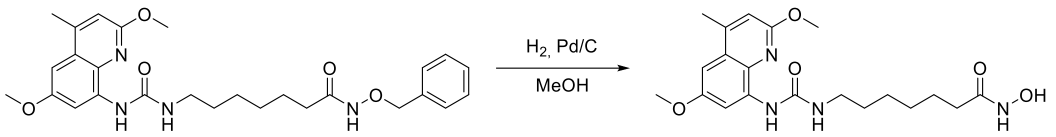

8 was dissolved in 50 mL of MeOH and 5 mol% of 10 % Pd/C was added to the reaction solution. The catalytic hydrogenation was performed for 5 h and reaction solution filtered through celite®. Afte the solvent was removed under reduced pressure, the product was purified by flash column chromatography (DCM/MeOH).

8 was synthesised on a 0.503 mmol scale with a yield of 64% (130 mg, 0.32 mmol) and obtained as a pink solid.

^{1}H NMR (300 MHz, DMSO-d_6) δ 10.34 (s, 1H), 9.94 (s, 1H), 9.59 (s, 1H), 8.66 (s, 1H), 8.13 (d, *J* = 2.7 Hz, 1H), 6.96 (d, *J* = 1.2 Hz, 1H), 6.92 (d, *J* = 2.7 Hz, 1H), 3.98 (s, 3H), 3.88 (d, *J* = 4.9 Hz, 5H), 2.71 – 2.55 (m, 3H), 1.94 (t, *J* = 7.3 Hz, 2H), 1.65 (p, *J* = 6.8 Hz, 2H), 1.58 – 1.42 (m, 2H), 1.34 (dq, *J* = 9.1, 4.6, 3.4 Hz, 2H).

^{13}C NMR (75 MHz, DMSO-d_6) δ 168.9, 159.3, 156.2, 155.7, 147.2, 134.1, 130.8, 125.0, 113.0, 106.2, 96.6, 75.8, 55.3, 52.6, 32.1, 27.5, 24.8, 18.4.

M.p.: 151.0°C; HPLC: R_t= 11.10 min, purity 95.3 %, **MS** (+APCI): 407 $[M+H]^+$.

3. NMR

*3.1. NMR of Compound **3***

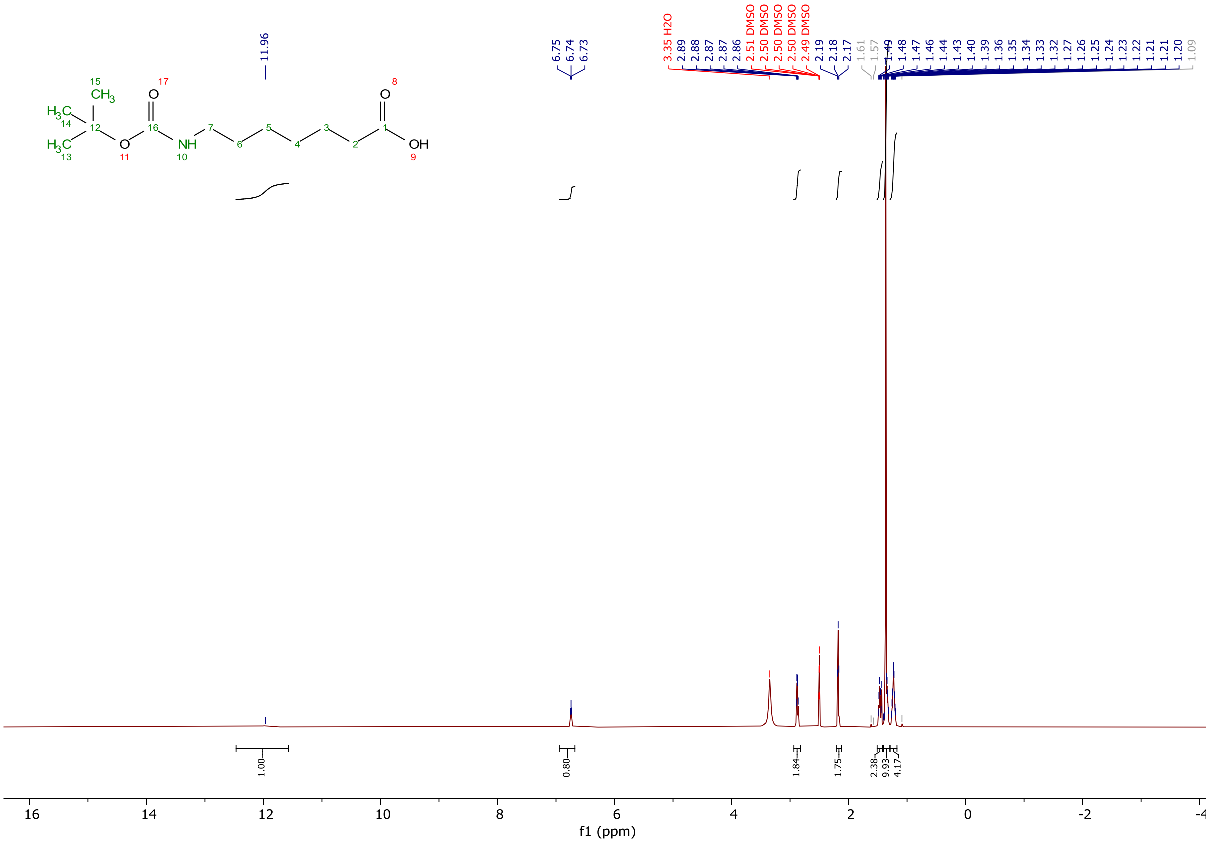

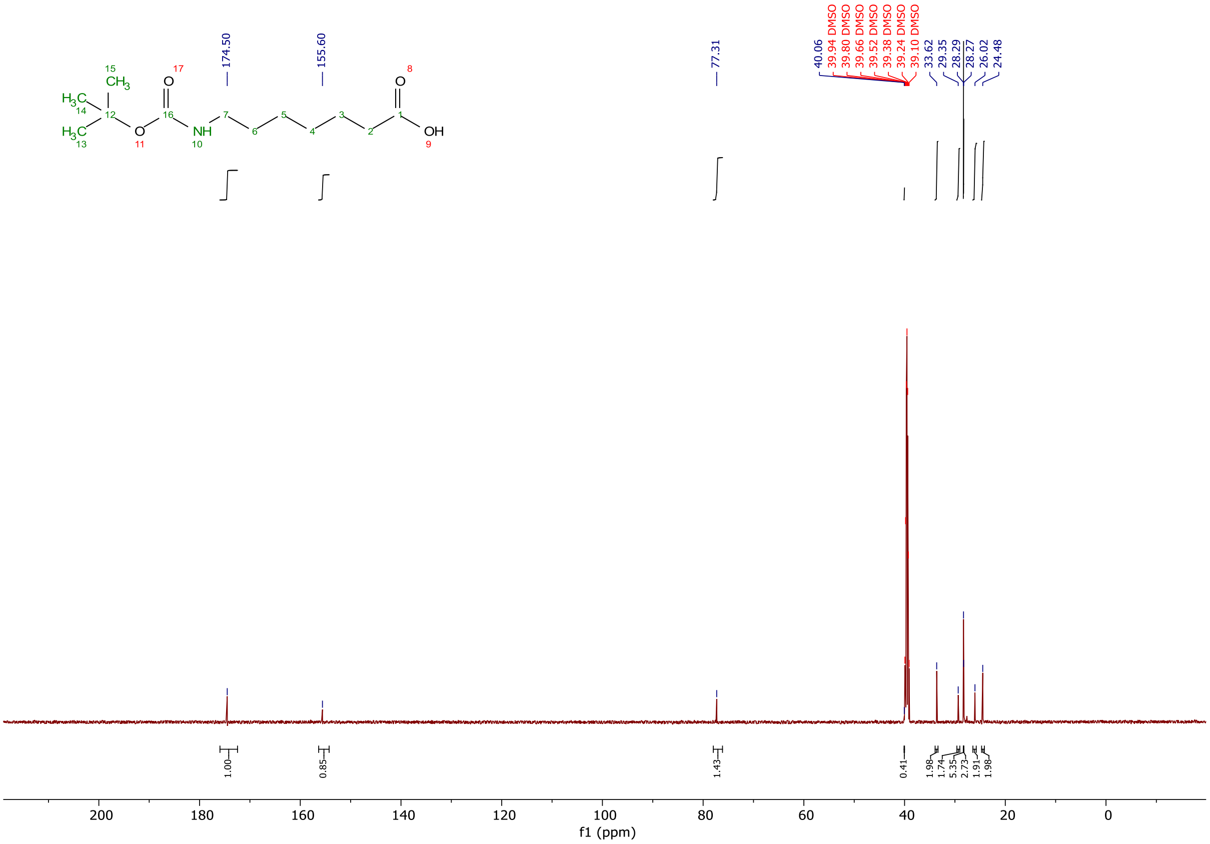

3.1. NMR of Compound **4**

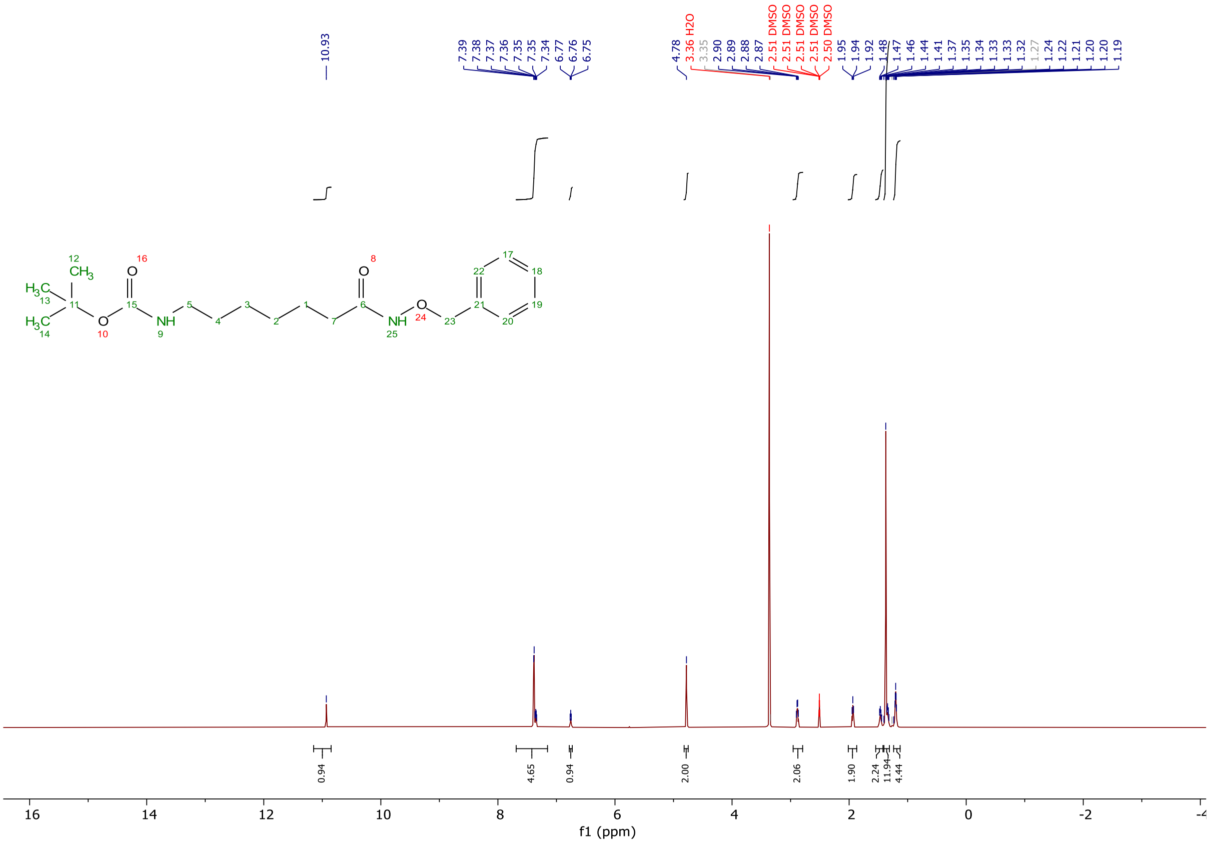

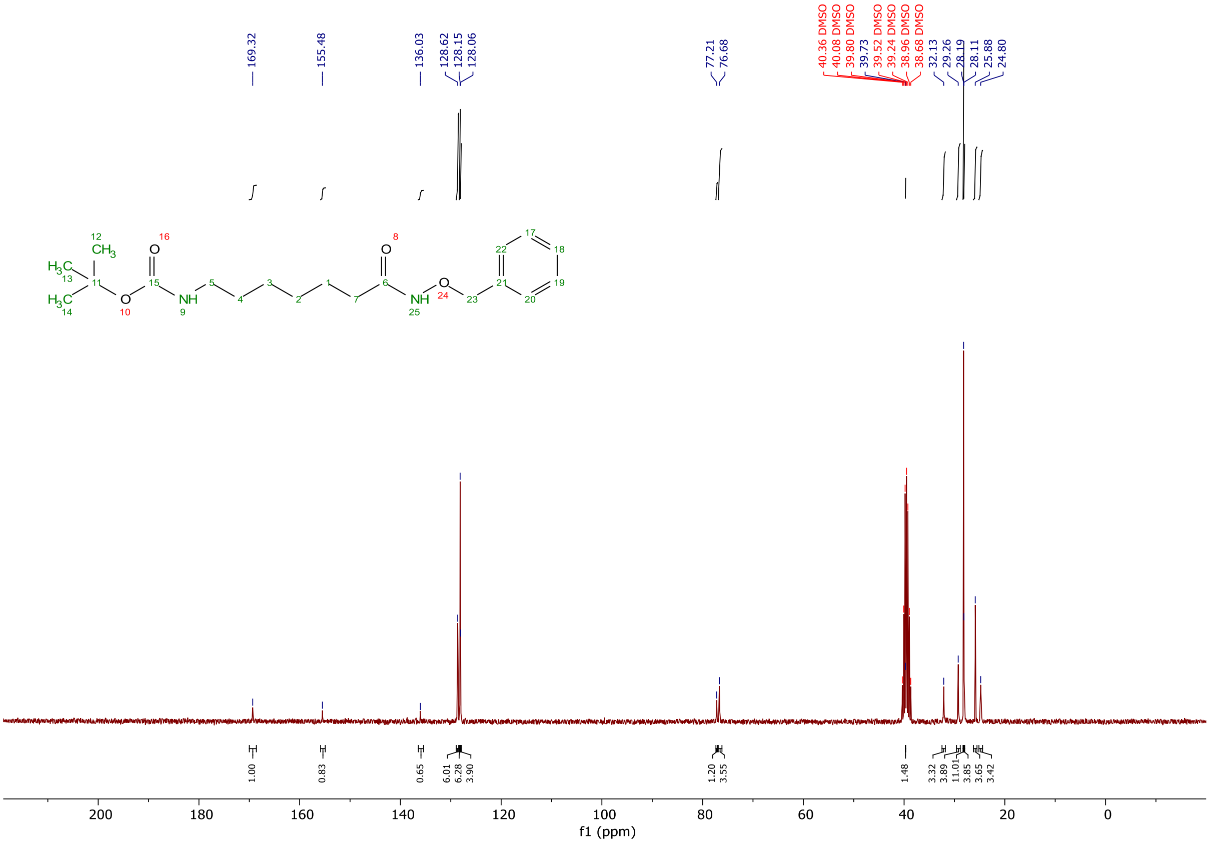

3.1. NMR of Compound ***5***

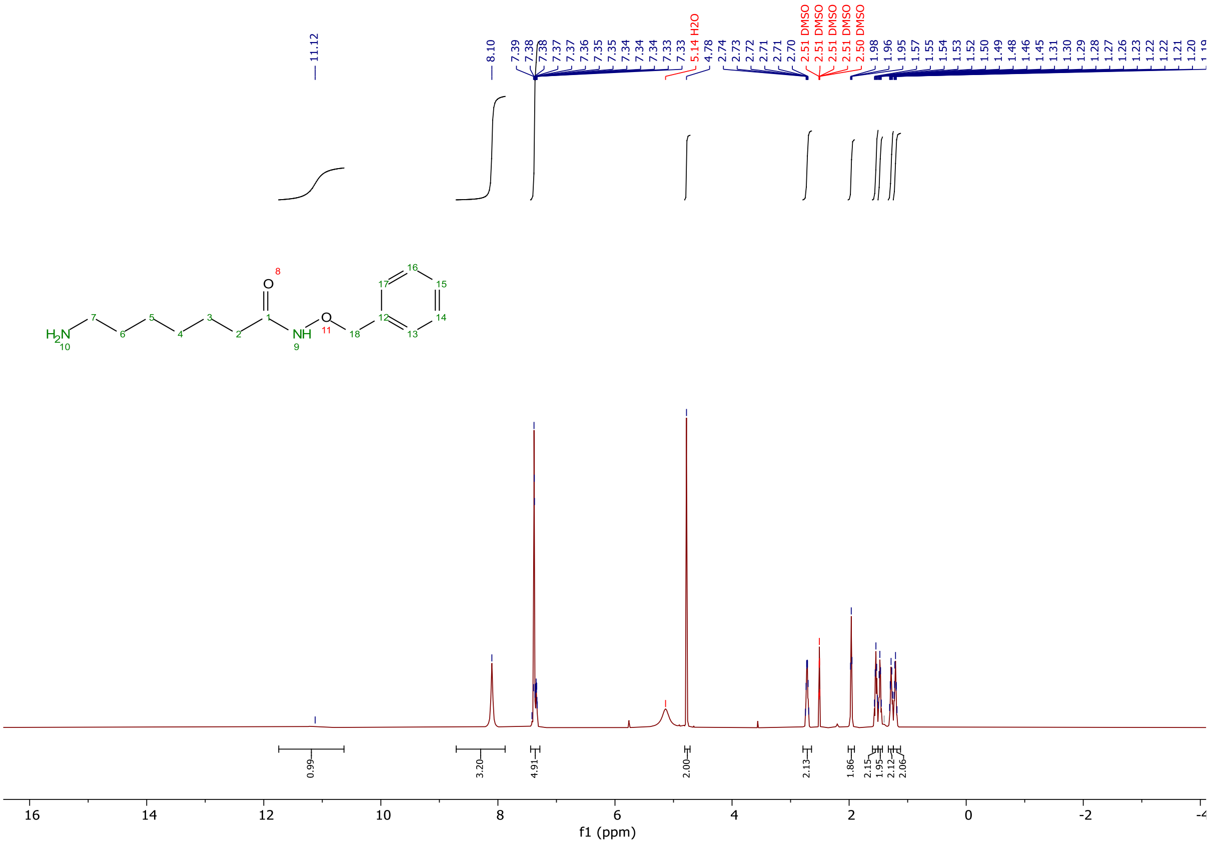

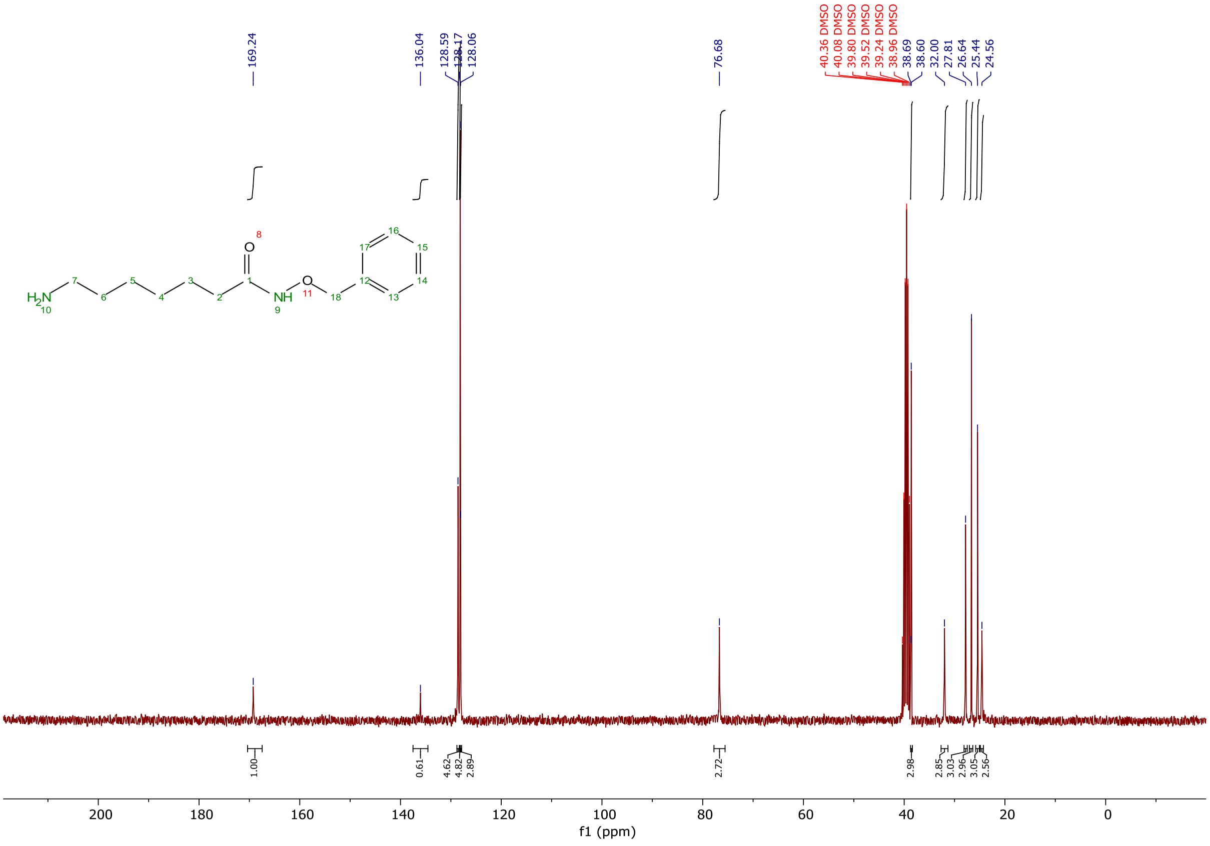

3.1. NMR of Compound **6**

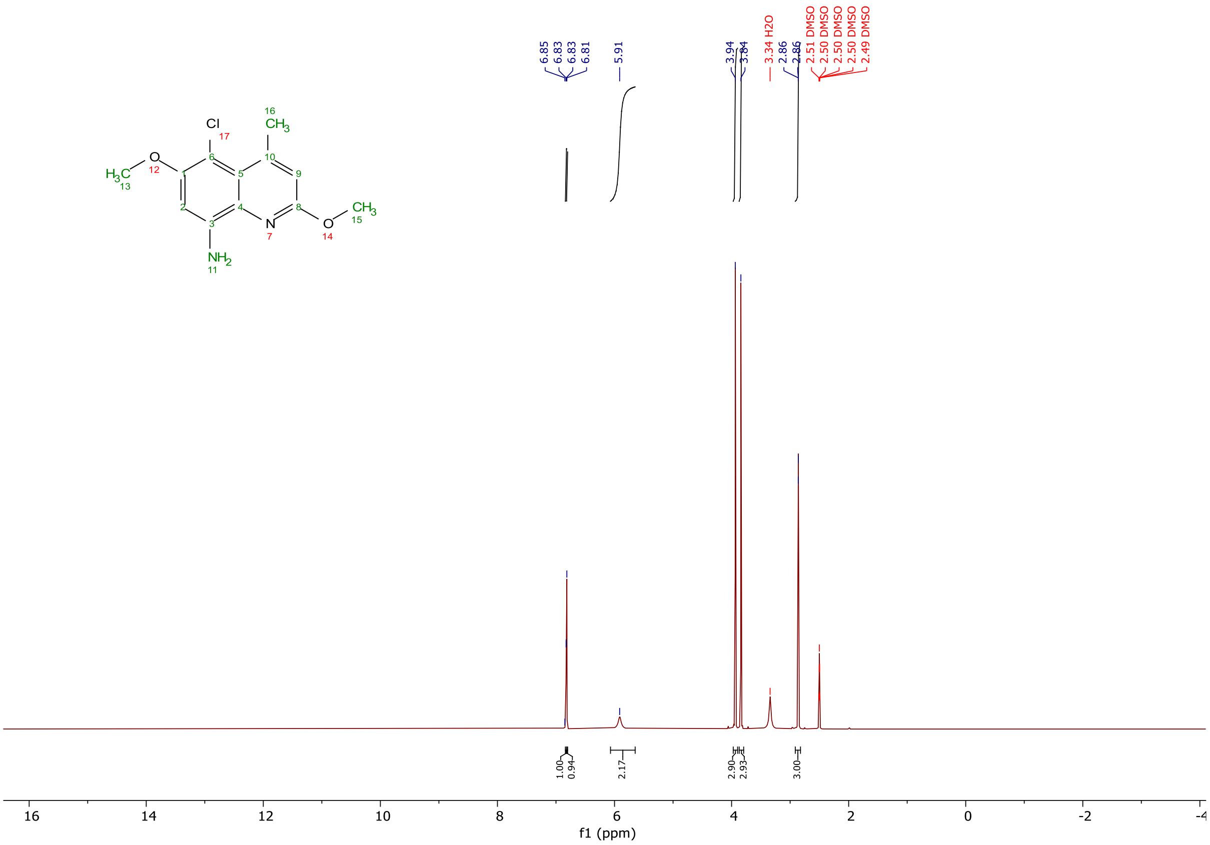

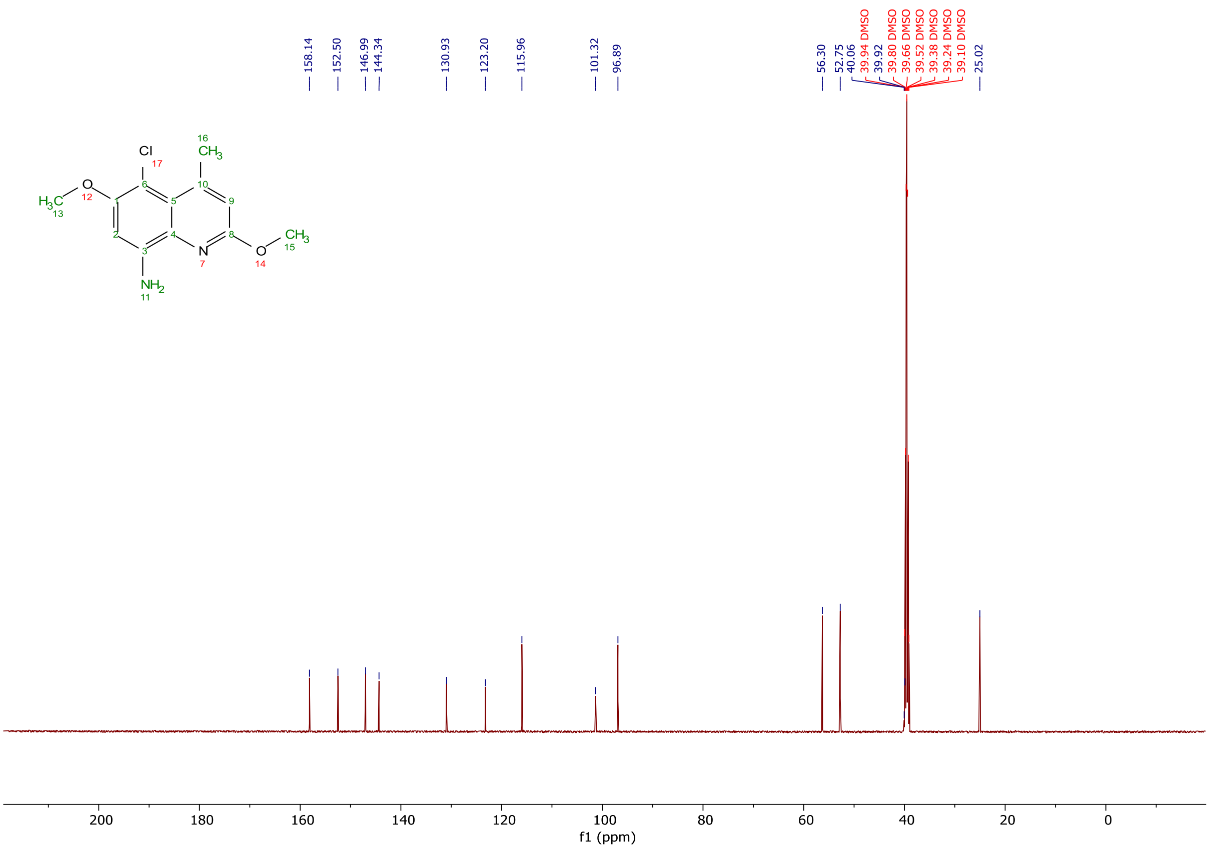

3.1. NMR of Compound **7**

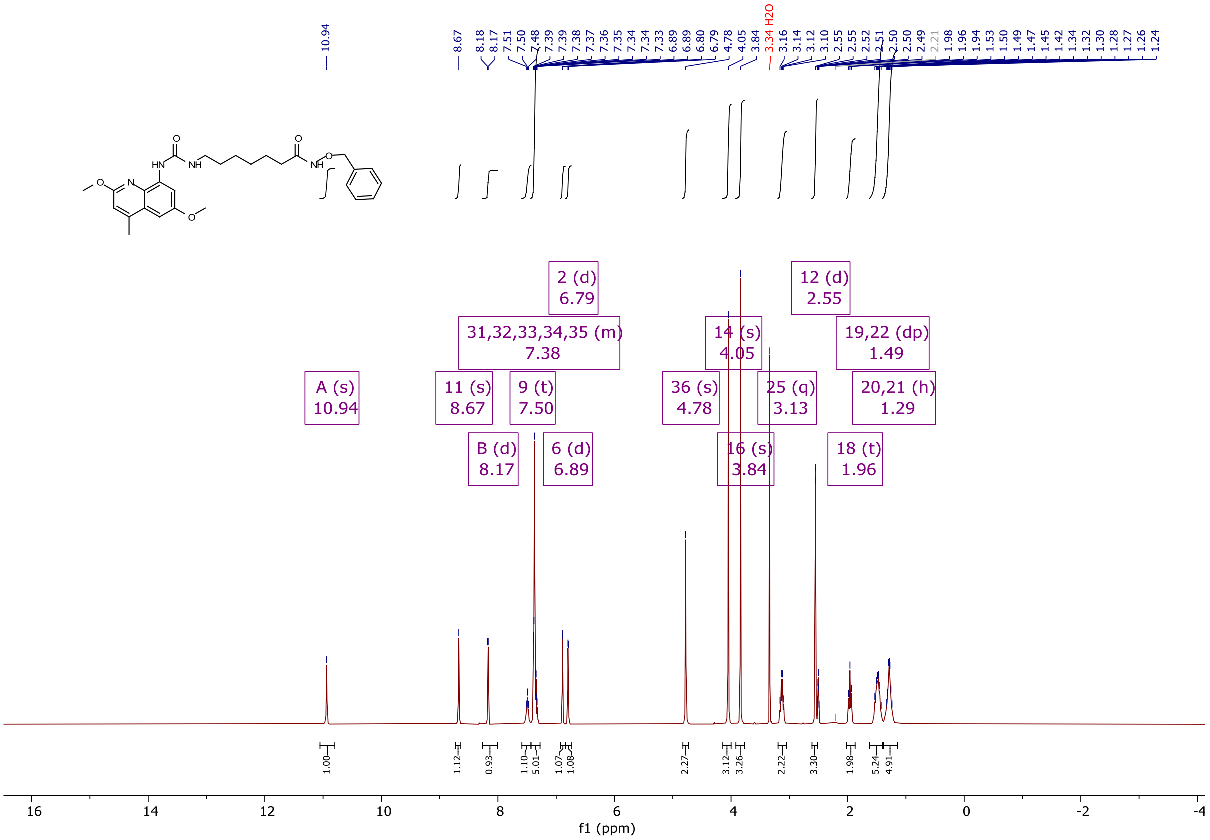

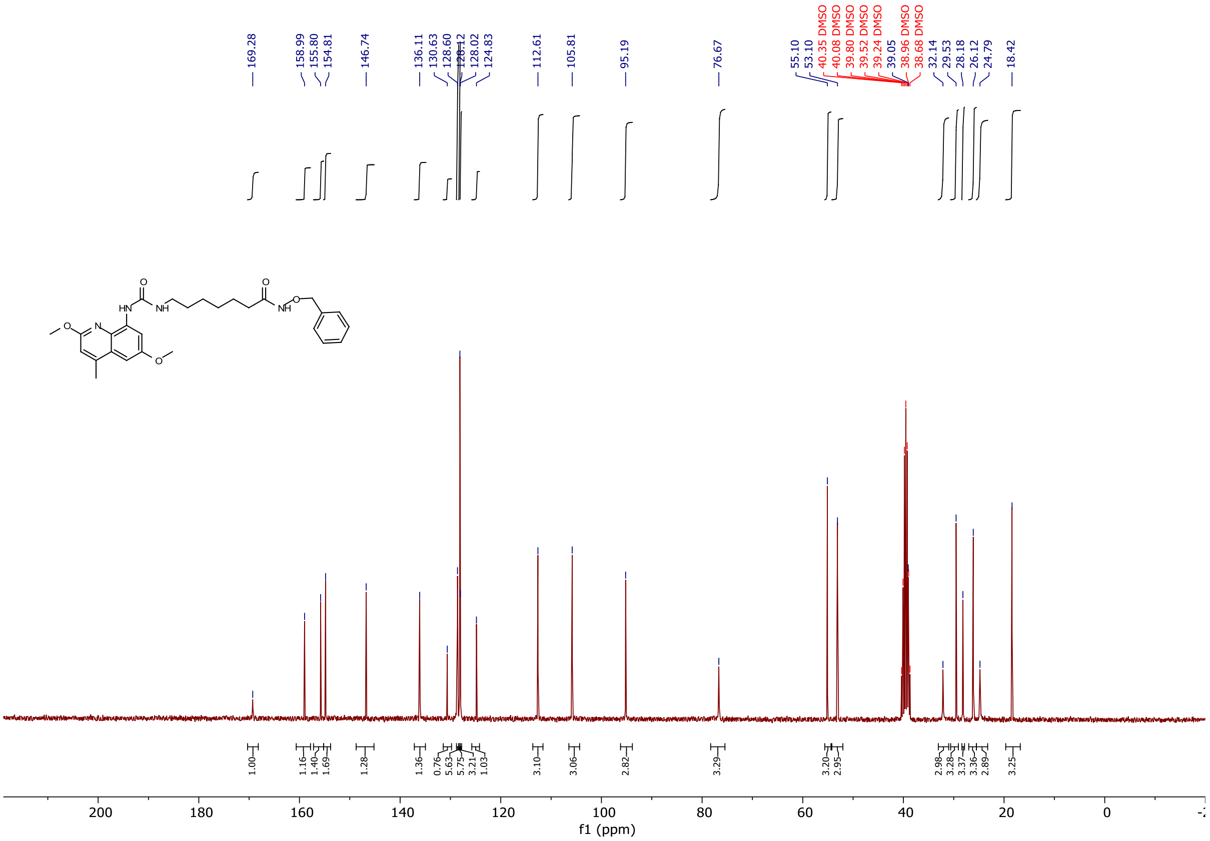

*3.2. NMR of Compound **8** (MPK544)*

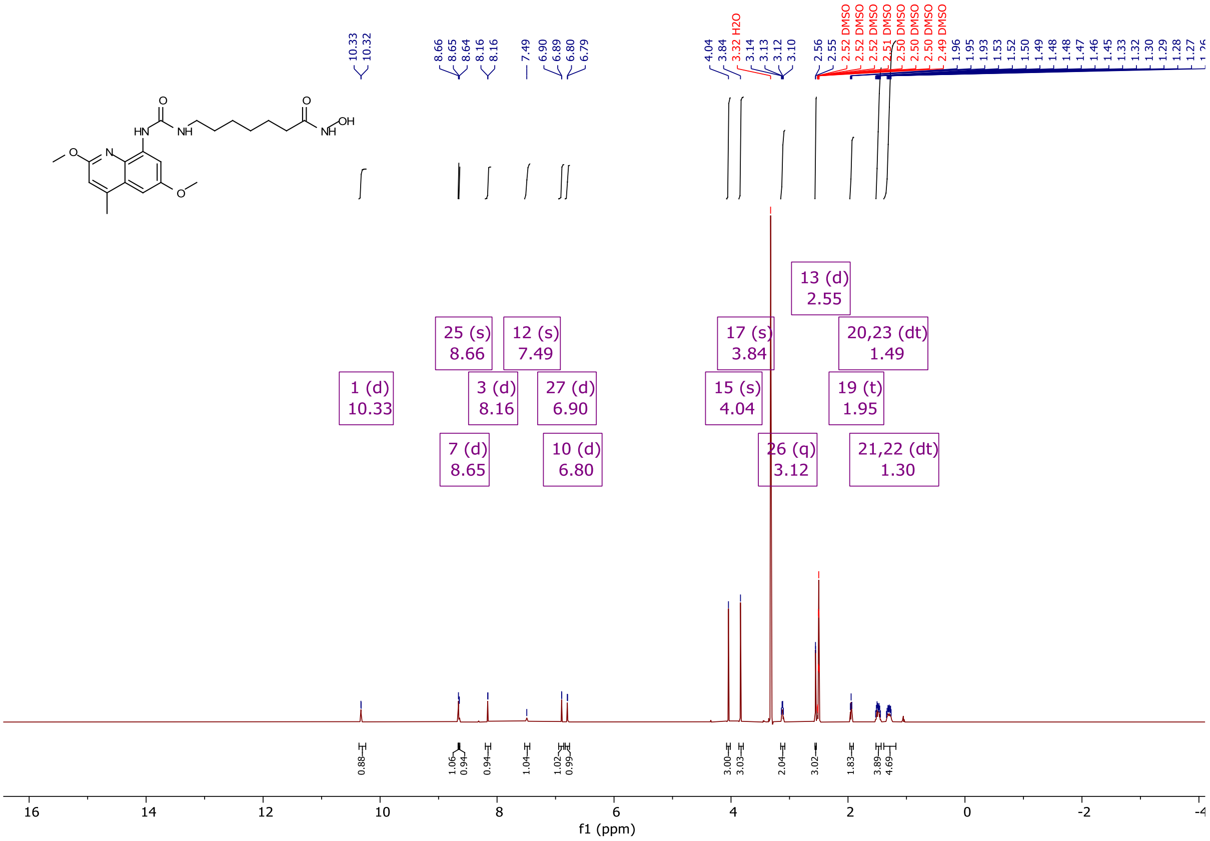

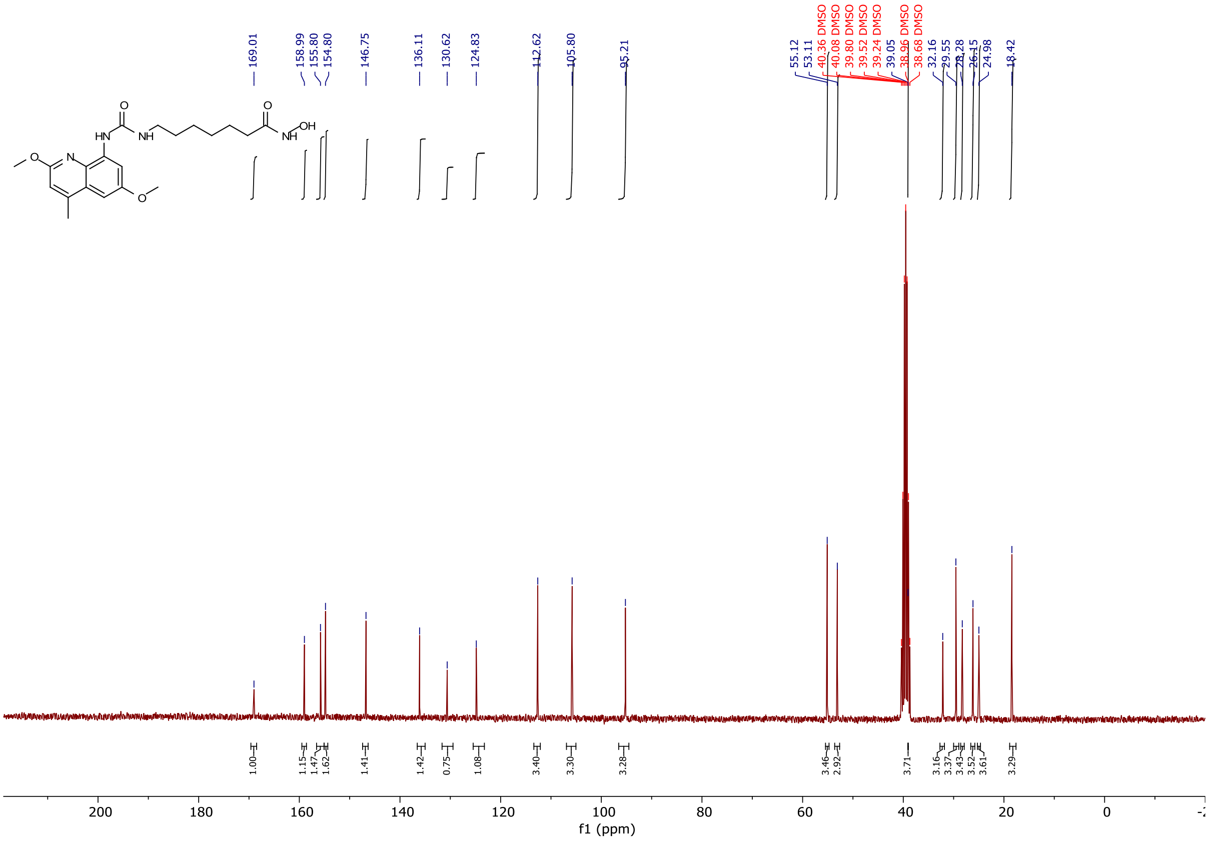

4. HDAC enzyme inhibition

All human recombinant enzymes were purchased from Reaction Biology Corp. (Malvern, PA, USA). The HDAC activity assay of HDAC2 (catalog nr. KDA-21-277), 4 (catalog nr. KDA-21-279), and 6 (catalog nr. KDA21-213) was performed in 96-well plates (Corning, Germany). Briefly 20ng of HDAC2 and, 17.5ng of HDAC6 and 2ng of HDAC4 per reaction were used. Recombinant enzymes were diluted in assay buffer (50 mM Tris-HCL, pH 8.0, 137 mM NaCl, 2.7 mM KCl, 1 mM MgCl2, and 1 mg/ml BSA). 80 µl of this dilution was incubated with 10 µl of different concentrations of inhibitors in assay buffer. After a 5 min incubation step the reaction was started with 10 µl of 300 µM (HDAC2), 150 µM (HDAC6) Boc-Lys(Ac)-AMC (Bachem, Germany) or 100 µM (HDAC4) BocLys(TFa)-AMC (Bachem, Germany). The reaction was stopped after 90 min by adding 100 µl stop solution (16mg/ml trypsin, 2 µM Panobinostat for HDAC2 and HDAC6, 2 µM CHDI0039 (kindly provided by the CHDI Foundation Inc., New York, USA) for HDAC4 in 50 mM Tris-HCL, pH 8.0, and 100 mM NaCl. 15 min after the addition of the stop solution the fluorescence intensity was measured at excitation of 355 nm and emission of 460 nm in a NOVOstar microplate reader (BMG LabTech, Offenburg, Germany).

5. **E-cadherin expression after 72h hours treatment with MPK544**

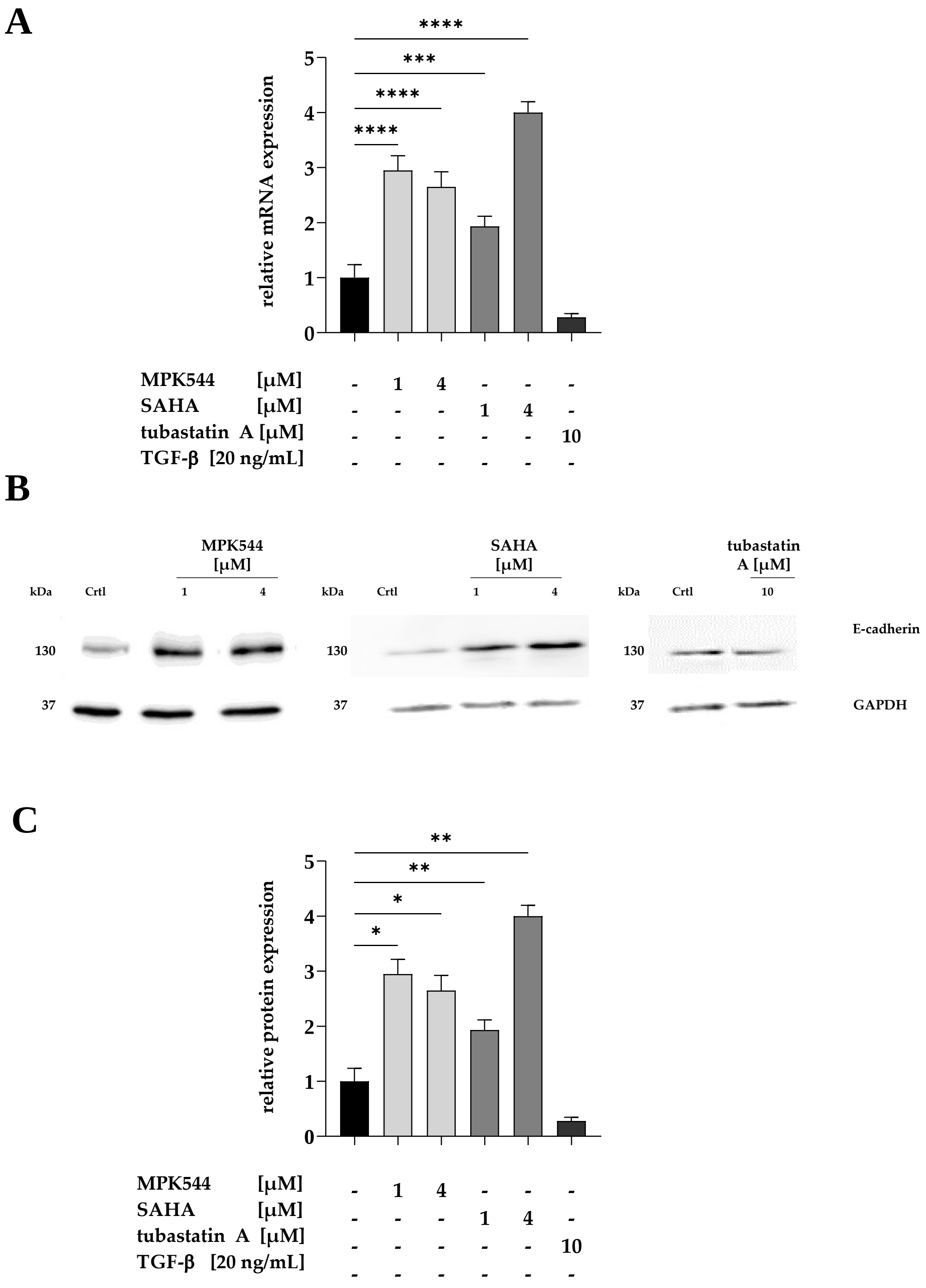

Supplementary Figure S1. Upregulation of E-cadherin expression in PANC-1 cells. PANC-1 cells were treated with MPK544, SAHA and tubastatin A for 72 hours. mRNA levels of E-cadherin in PANC-1 cells were analyzed by qRT-PCR (A) and at the protein level by western blot (B, C). Representative western blots are shown (B). Error bars indicate the standard errors of the mean (of at least three independent experiments, with * = $p \leq 0.05$; ** = $p \leq 0.01$; *** = $p \leq 0.001$; and **** = $p \leq 0.0001$).

6. IF-staining for E-cadherin in PANC-1 spheroids

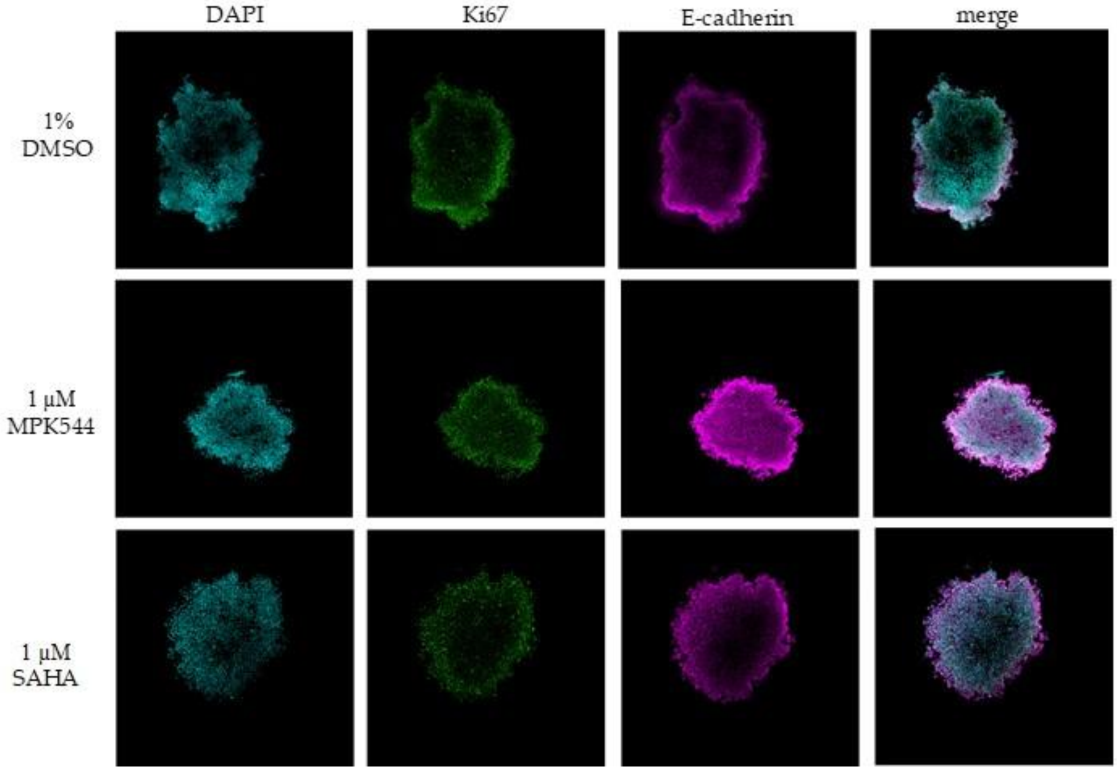

Supplementary Figure S2. E-cadherin expression in PANC-1 spheroids after 72h of treatment with MPK544 or SAHA. DAPI-staining of nuclei (405 nm); staining of the proliferation marker Ki67 (488 nm); staining of E-cadherin (647 nm); merging of all three channels. Imaging of spheroids with the CQ1 High-Content Spinning Disk System (Yokogawa Electric Corporation). IC_{50} values for PANC-1 spheroids are 2.17µM for SAHA and 2.20 µM for MPK544.

References

1. Hexahydro-2-(1H)-Azocinone. *Org. Synth.* **1985**.
2. Giovannini, A.; Savoia, D.; Umani-Ronchi, A. Organometallic ring-opening reactions of N-acyl and N-alkoxycarbonyl lactams. Synthesis of cyclic imines. *J. Org. Chem.* **1989**, *54*, 228–234, doi:10.1021/JO00262A048.
3. Ugwuegbulam, C.O.; Foy, J.E. Process for the preparation of anti-malarial drugs.
